# Does the use of pharmacotherapy interact with the effects of psychotherapy? A meta-analytic review

**DOI:** 10.1192/j.eurpsy.2023.2437

**Published:** 2023-08-03

**Authors:** Pim Cuijpers, Clara Miguel, Mathias Harrer, Marketa Ciharova, Eirini Karyotaki

**Affiliations:** 1 Department of Clinical, Neuro and Developmental Psychology, Amsterdam Public Health Research Institute, Vrije Universiteit Amsterdam, Amsterdam, The Netherlands; 2 International Institute for Psychotherapy, Babeș-Bolyai University, Cluj-Napoca, Romania; 3 Psychology & Digital Mental Health Care, Department of Health Sciences, Technical University Munich, Munich, Germany; 4 Department of Clinical Psychology & Psychotherapy, Friedrich-Alexander-University Erlangen-Nuremberg, Erlangen, Germany

**Keywords:** antidepressants, depression, meta-analysis, psychotherapy, major depressive disorder

## Abstract

**Background:**

It is not clear if there is an interaction between psychotherapy and pharmacotherapy. First, there may be no interaction at all, meaning that the effects of both are independent of each other. Second, antidepressants may reduce the effects of psychotherapy, and third, antidepressants may increase the effects of psychotherapy. We examined which of the three is correct.

**Methods:**

We conducted random effects meta-analyses of randomized trials comparing psychotherapies for adult depression with control conditions. The proportion of users of antidepressants was used as a predictor of the effect size in a series of meta-regression analyses, while adjusting for relevant moderators, such as type of control group and baseline severity.

**Results:**

We included 300 randomized controlled trials (353 comparisons between treatment and control; 32,852 participants). The main effect size of psychotherapy was *g* = 0.71 (95% CI: 0.64; 0.79) with high heterogeneity (*I*^2^ = 82; 95% CI: 80; 84). We found no significant association between the proportion of antidepressants users and effect size (*p* = .07). We did find a significant association with some other predictors, including the type of control group and risk of bias. The use of antidepressants was associated with higher response rates within the control conditions, but not with the relative effects of the treatments compared to the control groups.

**Discussion:**

We found support for the independent effects of psychotherapy and pharmacotherapy, which is good news from a clinical perspective. Apparently, patients can start with psychotherapy and do not have to be afraid that this will reduce the effects of the therapy.

## Introduction

Large meta-analyses with hundreds of studies have shown that psychotherapy [1] and pharmacotherapy [2] are effective in the treatment of depression. It has also been well-established that the combination of the two is more effective than either one alone [3]. Psychotherapy has been found to be more effective than pharmacotherapy at the longer term, although the combination of the two is still more effective [4].

It is not yet clear, however, whether there is an interaction between psychotherapy and pharmacotherapy. Theoretically, there are three ways in which pharmacotherapy can interact with psychotherapy. First, there may be no interaction at all. In that case, the effects of both treatments are completely independent of each other, meaning that the effect of combined treatment is simply the sum of the effects of psychotherapy plus the effects of pharmacotherapy. Second, the use of antidepressants may reduce the effects of psychotherapy. For example, there could be a ceiling effect, resulting in a reduction of the overall effects of psychotherapy. Third, the use of antidepressants may enhance the effects of psychotherapy. This may be the case because the strengths of each modality are promoted during combined treatment while the weaknesses of each modality are minimized [5, 6].

In order to examine which of these three possible ways of interaction is correct, randomized trials are needed that can assess the independent effects of psychotherapy, the independent effects of pharmacotherapy, and the independent effects of combined treatment. Such randomized trials need to include four arms, namely combined treatment, pharmacotherapy, psychotherapy, and a control condition (placebo). This design allows us to examine the effects of both treatments as well as the effects of the combined treatment. When these three treatment effects are known, it can be checked whether the effect of the combined treatment is the sum of the effects of the two separate treatments or that the sum of the effects of the single treatments is larger or smaller.

Unfortunately, only a few trials with these four arms have been conducted. In an earlier meta-analysis, we identified 11 trials with such a design [7]. We found that the effects of combined treatment versus placebo (*g* = 0.74) were indeed roughly the sum of the effects of psychotherapy (*g* = 0.37) and pharmacotherapy (*g* = 0.35). However, most of these studies were aimed at anxiety and not at depression, and each of these effect sizes had a rather broad confidence interval, making the conclusion that they do not interact uncertain.

Another way to explore a possible interaction between pharmacotherapy and psychotherapy is to examine in a meta-analysis whether there is an association between the proportion of patients in psychotherapy who also use pharmacotherapy and the outcome of the psychotherapy for depression. Many randomized trials exclude people who use antidepressants, while in other trials, 100% of all participants receiving psychotherapy also use antidepressants. In most trials, the proportion of antidepressant users is somewhere between 0 and 100%. If pharmacotherapy interacts with the effects of psychotherapy, it could be expected that there is an association between the proportion of antidepressants users and the effects of psychotherapy. To the best of our knowledge, no previous meta-analysis has examined this and we decided to do a meta-analysis focused on this question using our large database of randomized controlled trials on psychotherapy for depression [8].

## Methods

### Identification and selection of studies

The current study is part of a larger meta-analytic project on psychological treatments of depression that was registered at the Open Science Framework [9] and supplemental materials are available at the website of the project (www.metapsy.org). This database has been used in a series of earlier published meta-analyses [10]. The protocol for the current meta-analysis has been published in the Open Science Framework [11].

The studies included in the current study were identified through the larger, already existing database of randomized trials on the psychological treatment of depression. For this database, we searched four major bibliographical databases (PubMed, PsycINFO, Embase, and the Cochrane Library) by combining index and free terms indicative of depression and psychotherapies, with filters for randomized controlled trials. The full search strings are available at the website of the project (www.metapsy.org and docs.metapsy.org/databases). Furthermore, we checked the references of earlier meta-analyses on psychological treatments of depression. The database is updated every 4 months (from 1966 to September 1, 2022). All records were screened by two independent researchers and all papers that could possibly meet inclusion criteria according to one of the researchers were retrieved as full-text. The decision to include or exclude a study in the database was also done by the two independent researchers, and disagreements were resolved through discussion.

For the current meta-analysis, we selected randomized controlled trials in which psychological treatments of depression were compared with an inactive control group (waiting list, care-as-usual, pill placebo, other). We selected only trials in which the proportion of participants in the trials who used antidepressants was reported. Studies should specifically report the use of antidepressants, and more general descriptions of medication use were not sufficient for inclusion. We also included trials in which all participants received antidepressants (comparisons between combined treatment and pharmacotherapy only), because these were considered as 100% use of antidepressants.

Depression could be defined as meeting criteria for a depressive disorder according to a diagnostic interview or as a score above the cut-off on a validated self-report depression measure. We only included individual, group, telephone, and guided self-help interventions. Interventions without any human interaction (unguided self-help) were not included, because these are significantly less effective than other formats [12–14]. Inpatient settings were not included in these analyses [15]. We also excluded studies in children and adolescents because psychological treatment is significantly less effective in these age groups [16].

### Quality assessment and data extraction

We assessed the validity of included studies using four criteria of the “Risk of bias” (RoB) assessment tool, version 1, developed by the Cochrane Collaboration [17]. We used version 1 of this tool because this meta-analysis is included in the broader meta-analytic project of psychological treatments of depression [18]. The RoB tool assesses possible sources of bias in randomized trials, including the adequate generation of allocation sequence; the concealment of allocation to conditions; the prevention of knowledge of the allocated intervention (masking of assessors); and dealing with incomplete outcome data (this was assessed as positive when intention-to-treat analyses were conducted, meaning that all randomized patients were included in the analyses). We considered trials as having a low risk of bias when they scored positive on all four domains.

We also coded participant characteristics (diagnostic method for inclusion; recruitment method; target group; mean age; proportion of women); characteristics of the psychological treatments (type of therapy; format; number of sessions), as well as general characteristics of the studies (type of control; publication year; country). The details of these characteristics can be found on the website of the project (docs.metapsy.org/databases/depression-psyctr/). For the current study, we also extracted the proportion of participants who used antidepressants. Because studies with more users of antidepressants could be aimed at more severely depressed populations, we also calculated baseline severity. We converted the most common depression measures (BDI-I, BDI-II, MADRS, PHQ-9, EPDS) to the HDRS-17 [19], using established conversion methods [20–22].

All assessments of all characteristics were conducted by two independent researchers, and disagreements were solved through discussion.

### Outcome measures

For each comparison between a treatment and a control condition, the effect size indicating the difference between the two groups at post-test was calculated (Hedges’ *g*). Because some studies were expected to have relatively small sample sizes, we corrected the effect size for small sample bias. When means and standard deviations were not reported in a study, we used change scores. If these were not reported either, we converted binary outcomes to Hedges’ *g* or used other statistics (e.g., *p*-value, *t*-value) to calculate the effect size.

### Meta-analyses

The meta-analyses were conducted using the metapsyTools package in R (version 4.1.1) [23], and Rstudio (version 1.1.463 for Mac). The metapsyTools package was specifically developed for the meta-analytic project of which this study is a part. This package imports the functionality of the meta [24], metafor [25], and dmetar packages [26].

We calculated pooled effect sizes in several ways, as implemented in the metapsyTools package, to explore if different pooling methods resulted in different outcomes. In our main model, all effect size data available for comparison in a specific study were aggregated within that comparison first. These aggregated effects were then pooled across studies and comparisons. An intra-study correlation coefficient of *ρ* = .5 was assumed to aggregate effects within comparisons.

We conducted several other analyses to examine whether these main outcomes are robust. First, we estimated the pooled effect using a three-level “correlated and hierarchical effects” (CHE) model [27]; parameter tests and confidence intervals of which were also calculated using RVE to guard against model misspecification. We assumed an intra-study correlation of *ρ* = .5 for this model. Second, we pooled effects while excluding outliers, using the “nonoverlapping confidence intervals” approach, in which a study is defined as an outlier when the 95% confidence interval (CI) of the effect size does not overlap with the 95% CI of the pooled effect size [28]. Third, we pooled effects while excluding influential cases as defined by the diagnostics in Viechtbauer and Cheung [29]. Fourth, we calculated the effect when only the smallest or largest effect in each study was considered. Fifth, we estimated the pooled effect using only studies with a low risk of bias. We also used three different methods to assess and adjust for potential publication bias [28, 30]: Duval and Tweedie’s trim and fill procedure [31], Rücker’s “Limit meta-analysis method” [32], and a step function selection model [33, 34].

A random-effects model was assumed for all analyses. Between-study heterogeneity variance (components) was estimated using restricted maximum likelihood. For models not fitted using RVE, we applied the Knapp–Hartung method to obtain robust confidence intervals and significance tests of the overall effect [35]. As a test of homogeneity of effect sizes, we calculated the *I*^2^-statistic and its 95% CI, which is an indicator of heterogeneity in percentages [36]. For the three-level model, we calculated a multilevel extension of *I*^2^, which describes the amount of total variability attributable to heterogeneity within studies (level 2) and heterogeneity between studies (level 3) [37]. Because *I*^2^ cannot be interpreted as an absolute measure of the between-study heterogeneity, we also added the prediction interval (PI) to the main analyses, which indicates the range in which the true effect size of 95% of all populations will fall [38, 39]. We estimated the number-needed-to-treat (NNT) for depression using the formulae provided by Furukawa [40] (assuming the control group’s event rate at a conservative 17%; [41]).

We conducted a univariate meta-regression analysis to examine the association between the effect size and the proportion of participants using an antidepressant at baseline. Then we conducted a multivariable meta-regression analysis in which we also added other major characteristics of the participants (including baseline severity), the interventions, and the studies.

We conducted additional analyses in which we compared studies in which 100% of participants used antidepressants, and studies in which no participants used them. Then we conducted again a multivariable meta-regression analysis with a dummy variable indicating 0% or 100% participants using antidepressants, and adjusting for the other major characteristics of the studies.

As sensitivity analyses, we estimated the response rates (a 50% reduction of depressive symptoms compared to baseline) of the therapy and control conditions separately. We estimated the response rates using a validated method using the baseline means, the post-test means, the post-test *SD*, and *N* [42]. When more than one outcome measure was reported, we selected the outcome according to an algorithm that has been used in previous meta-analyses [3]. Then we conducted a univariate meta-regression to test whether the proportion users of antidepressants were associated with the proportion responders, in the treatment groups and in the control groups. Then we conducted a multivariable meta-regression analysis with the proportion antidepressant users (separately for the treatment and control groups) and the predictors that were found to be significantly associated with the effect size in the main multivariable meta-regression analysis.

## Results

### Selection and inclusion of studies

**Figure 1.**
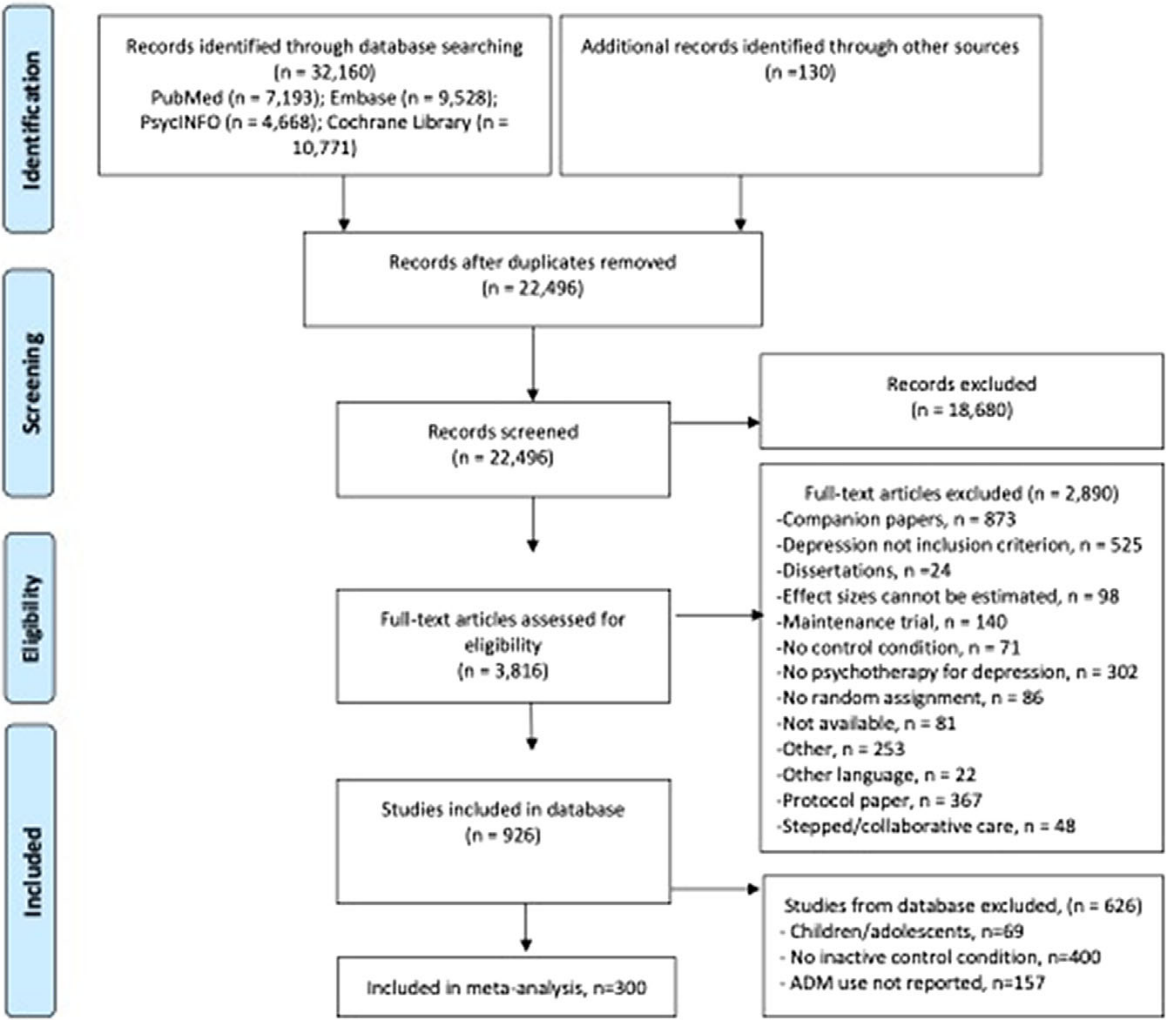
Flowchart of the inclusion of studies.

After examining a total of 32,290 records 22,496 after the removal of duplicates, we retrieved 3,816 full-text papers for further consideration. We excluded 3,236 of the retrieved papers. The PRISMA flowchart describing the inclusion process, including the reasons for exclusion, is presented in Figure 1. A total of 300 randomized controlled trials (with 353 comparisons between a treatment and a control group) met the inclusion criteria for this meta-analysis. The references to the included studies are given in Supplementary Appendix A.

### Characteristics of included studies

A summary of key characteristics of the 300 included studies is presented in Supplementary Appendix B. An overview of aggregated characteristics is given in Table 1. In the trials, 32,852 patients participated, 17,405 in the intervention, and 15,447 in the control conditions.

**Table 1.** Aggregated characteristics of the included studies

			*N* (*M*)	% (*SD*)
*Trial characteristics (k = 300)*				
Antidepressant use (*M*, *SD*)			(41.1)	(38.8)
Mean age (*M*, *SD*)			(44.6)	(14.0)
Proportion women (*M*, *SD*)			(0.72)	(0.20)
Diagnosis		Diagnosed depressive disorder	189	63.0
		Above cut-off	96	32.0
		Subthreshold depression	15	5.0
Target group		Adults	136	45.3
		General medical patients	65	21.7
		Older adults	25	8.3
		Perinatal depression	25	8.3
		College students	13	4.3
		Other specific target group	36	12.0
Recruitment		Clinical	101	33.7
		Community	120	40.0
		Other	79	26.3
Control		Care-as-usual	121	40.3
		Other control	38	12.7
		Pharmacotherapy	51	17.0
		Waitlist	90	30.0
Country		US	99	33.0
		Europe	74	24.7
		Australia	14	4.7
		Canada	18	6.0
		East Asia	32	10.7
		UK	36	12.0
		Other	27	9.0
Low risk of bias		Sequence generation	186	62.0
		Allocation concealment	151	50.3
		Blinded assessment^a^	278	92.3
		Intention to treat	200	66.3
Total risk of bias		0	9	3.0
		1	56	18.7
		2	57	19.0
		3	71	23.7
		4	107	35.7
*Intervention characteristics (k = 353)*				
Therapy		Cognitive behavior therapy	175	49.6
		Third wave therapy	30	8.5
		Behavioral activation therapy	24	6.8
		Psychodynamic therapy	10	2.8
		Interpersonal psychotherapy	31	8.8
		Life review therapy	10	2.8
		Problem-solving therapy	20	5.7
		Other	53	15.0
Format		Individual	151	42.8
		Group	101	28.6
		Guided self-help	56	15.9
		Telephone	17	4.8
		Other/mixed	28	7.9
*N* sessions (*M*, *SD*)			(10.3)	(8.0)

a This includes 154 studies that used only self-report measures.

In 189 trials, participants met the criteria for a depressive disorder according to a diagnostic interview, 96 trials used a cut-off score on a self-report measure to include participants, and 15 trials focused on subthreshold depression (clinically relevant symptoms but no depressive disorder). In 101 trials participants were recruited through clinical referrals, 120 conducted recruitment through the community, and 79 used other recruitment methods. A total of 136 studies were aimed at adults in general, 65 on patients with comorbid general medical disorders, 25 on older adults, 25 on women with perinatal depression, 13 on college students, and 36 on other specific target groups.

In 121 studies, usual care was used as the control group, 90 used a waitlist control group, in 51 studies all participants received pharmacotherapy (also in the control group), and the 38 remaining studies used another inactive control group. Ninety-nine studies were conducted in the US, 74 in Europe, and the remaining 127 in other countries.

The 300 trials included 353 interventions arms that were compared with a control group. A total of 175 of the intervention arms examined CBT, while all other therapies were examined in 30 or less trials. Totally, 151 interventions used an individual format, 101 had a group format, 56 had a guided self-help format, 17 delivered the intervention through the telephone, and the remaining 28 studies had a mixed format. The mean number of sessions was 10.3.

In total, 186 of the 300 studies reported an adequate sequence generation; 151 reported allocation to conditions by an independent party; 124 reported using blinded outcome assessors, while 154 used only self-report outcomes. In 200 studies, intent-to-treat analyses were conducted. In total, 107 studies met all criteria for low risk of bias, 128 studies met 2 or 3 criteria, and 65 met only one or none of the criteria.

### Main effects of the psychological treatments

The pooled effects of the psychotherapy conditions compared with the control conditions can be found in Table 2. The primary analyses, in which effect sizes were pooled within a study before pooling across studies was *g* = 0.71 (95% CI: 0.64; 0.79) with high heterogeneity (*I*^2^ = 82; 95% CI: 80; 84) and a wide prediction interval (−0.48; 1.91). The effect size corresponded with an NNT of 4.26. All sensitivity analyses resulted in a significant effect, although the effects were considerably smaller when only studies with a low risk of bias were included (*g* = 0.45; 95% 0.36; 0.54). The effect sizes were also considerably smaller after adjustment for publication bias (*g* ranged from 0.25 to 0.58). Heterogeneity was high in all analyses, except when 105 outliers were removed (*I*^2^ = 23; 95% CI: 9; 35). The prediction intervals were very wide in all analyses.

**Table 2.** Pooled effects of psychotherapies for adult depression

	*k*	*g*	95% CI	*I*^2^	95% CI	95% PI	NNT
All comparisons (combined)	353	0.71	0.64; 0.79	82	80; 84	−0.48; 1.91	4.26
One ES/study (lowest)	300	0.63	0.55; 0.71	82	80; 84	−0.53; 1.78	4.95
One ES/study (highest)	300	0.72	0.64; 0.80	83	81; 84	−0.49; 1.93	4.2
Outliers removed	248	0.62	0.58; 0.65	23	9; 35	0.39; 0.84	5.04
Influence analysis	343	0.62	0.56; 0.68	74	72; 77	−0.22; 1.45	5.05
Only risk of bias > 3	115	0.45	0.36; 0.54	73	68; 78	−0.3; 1.20	7.31
Three-level model (CHE)	410	0.68	0.61; 0.76	88	–	−0.51; 1.88	4.49
*Publication bias correction*							
– Trim-and-fill method	457	0.38	0.29; 0.48	88	87; 89	−1.45; 2.21	8.82
– Limit meta-analysis	353	0.25	0.14; 0.36	93	–	−0.95; 1.45	14.23
– Selection model^l^	353	0.58	0.47; 0.70	88	–	−0.88; 2.05	5.38

Abbreviations: CHE, “correlated and hierarchical effects” model; CI, confidence interval; ES, effect size; *g,* Hedges’ *g*; *I*^2^, level of heterogeneity; *k,* number of comparisons; NNT, number-needed-to-treat; PI, prediction interval.

### The association between the proportion users of antidepressants and the effects of psychotherapy

The univariate meta-regression analyses in which we entered the proportion of users of antidepressants as a predictor did not result in a significant association with the effect size (*p* = .07; Table 3). We entered the other main characteristics of the studies in a multiple meta-regression model, but the association between the proportion of antidepressant users and the effect size remained nonsignificant (*p* = .95). We did find several other significant predictors: IPT was significantly less effective than other therapies, that waitlist control groups resulted in larger effect sizes; countries outside Europe, North America, Australia and East Asia were associated with larger effect sizes; and studies with low risk of bias were associated with smaller effect sizes. We conducted another meta-regression analysis in which we only kept the significant predictors from the main multiple model, but the association between antidepressant use and the effect size remained nonsignificant (*p* = .96).

**Table 3.** Regression models of antidepressant use in trials comparing psychotherapy with control groups

		*k*	Coef.	SE	*p*	Coef.	SE	*p*	Coef.	SE	*p*
ADM use (continuous)		354	−0.02	0.00	0.07	−0.00	0.00	0.95	−0.00	0.00	0.96
Therapy	Third wave	30				Ref.					
	Behav act.	24				−0.03	0.19	0.88	−0.06	0.18	0.74
	CBT	175				−0.09	0.14	0.54	−0.11	0.13	0.39
	IPT	31				−0.51	0.18	*0.01*	−0.45	0.17	*<0.01*
	Probl. solv.	20				0.10	0.20	0.62	0.05	0.19	0.79
	Life rev.	10				0.38	0.28	0.17	0.31	0.25	0.21
	Dynamic	10				−0.07	0.25	0.78	−0.00	0.23	1.00
	Other	54				−0.28	0.16	0.07	−0.36	0.15	*0.02*
Control	Usual care	128				Ref.					
	Other control	44				−0.09	0.12	0.44	−0.21	0.11	0.06
	Pharmacoth	56				−0.23	0.15	0.13	−0.17	0.14	0.22
	Waitlist	126				0.26	0.10	*<0.01*	0.17	0.09	*0.05*
Year (continuous)						0.00	0.00	0.53			
Format	Group	101				Ref.					
	GSH	56				−0.19	0.12	0.12			
	Individual	152				−0.06	0.10	0.56			
	Telephone	17				0.09	0.18	0.63			
	Other/mixed	28				−0.12	0.15	0.40			
Number of sessions (continuous)		354				−0.01	0.00	0.12			
Country	Australia	16				Ref.					
	Canada	21				−0.06	0.23	0.80	−0.03	0.22	0.89
	East Asia	34				0.18	0.22	0.43	0.12	0.20	0.54
	Europe	86				−0.13	0.19	0.49	−0.13	0.18	0.47
	UK	37				−0.11	0.21	0.59	−0.08	0.20	0.68
	US	124				−0.09	0.19	0.64	0.01	0.18	0.96
	Other	36				0.63	0.23	*0.01*	0.66	0.20	*<0.01*
Mean age (continuous)		354				0.01	0.00	0.10			
Proportion women (continuous)		354				0.36	0.21	0.08			
Recruitment	Clinical	113				Ref.					
	Community	153				0.04	0.10	0.73			
	Other	88				0.10	0.13	0.47			
Diagnosis	Depressive dis	220				0.15	0.09	0.09			
	Cut-off	188				Ref.					
	Subthreshold	16				0.17	0.18	0.34			
Target group	Adults	164				Ref.					
	Gen med	70				−0.05	0.14	0.72			
	Older adults	29				−0.34	0.21	0.10			
	Other	45				0.23	0.13	0.07			
	Perinatal	27				0.07	0.20	0.72			
	Students	19				0.35	0.20	0.09			
Low RoB	Low versus other	354				*−0.26*	*0.08*	*<0.01*	−0.29	0.07	*<0.001*

*Note: In the first model, only the use antidepressants (ADM) was entered as a predictor; in the second model, all available characteristics were entered simultaneously; in the third model only predictors were retained that were significant in the second model.*

Abbreviations: ADM, antidepressant medication; Behav act, behavioral activation; CBT, cognitive behavior therapy; Coef, coefficient; Dis, disorder; Dynamic, psychodynamic therapy; Gen med, general medical patients; GSH, guided self-help; IPT, interpersonal psychotherapy; *k,* number of comparisons; *p, p*-value; perinatal, perinatal depression; pharmacoth, pharmacotherapy; Probl. solv, problem-solving therapy; RoB, risk of bias; SE, standard error; UK, United Kingdom; US, United States.

*p*-values indicate statistical significance.

Because we did find a trend (*p* = .07) suggesting that antidepressant use may be associated with a smaller effect size, we explored this association further in post hoc analyses. In the meta-regression analyses, we found that the effect size was significantly associated with the type of control group, with waitlist control groups having a significantly larger effect size. However, studies with 100% of antidepressant users by definition did not include waitlist control conditions. We, therefore, conducted another univariate meta-regression analysis in which we excluded the studies with a waitlist control group. We found that in this subset there was no trend anymore, suggesting that antidepressant use was not associated with the effect size in this analysis (coefficient: −0.00; SE = 0.00; *p* = .71).

We conducted one more multiple meta-regression analysis in which we also included baseline severity (HAMD-17 and other baseline measures converted to the HAMD-17). This is an important variable, because it can be assumed that patients receiving antidepressants often suffer from more severe depression than patients not receiving antidepressant. We examined this in a separate analysis because baseline severity was only available for a limited number of comparisons (*k* = 174). As can be seen in Supplementary Appendix C, baseline severity was not significant in these analyses either (*p* = .95).

We also conducted a sensitivity analysis limiting the analyses to studies in which 100% of participants received antidepressants or in which 0% of the participants used antidepressants. So, we left out the studies in which only a proportion of the participants used antidepressants and used a dummy variable indicating zero or 100% antidepressant use. We first conducted a meta-regression analysis with this dummy as the only predictor and did find that this was significantly associated with the effect size (*p* = .03; Supplementary Appendix D). After adjustment for all characteristics of the studies, the association between the effects and antidepressant use was not significant (*p* = 1.00), also not when we included baseline severity as a predictor in the analyses (*p* = .66). We also conducted a sensitivity analysis, excluding waitlist control groups. Because the studies with 100% antidepressant users did not include waitlist control groups by design, we also conducted a univariate regression analysis with the dummy variable, while excluding waitlist control groups. Again, we found no indication that the effect size was associated with the dummy variable (*p* = .44).

### Response rates

The overall response rate for the treatment groups was 0.43 (95% CI: 0.41; 0.46; *I*^2^ = 79; 95% CI: 77; 81). The association between antidepressant use and the response rates within the treatment groups was not significant (*p* = .33), also not after excluding waitlist control groups (*p* = .19), and after adding the study characteristics as predictors, including (*p* = 1.00) or excluding baseline severity (*p* = .24).

The response rate within in the control groups was 0.22 (95% CI: 0.20; 0.23; *I*^2^ = 74; 95% CI: 71; 77). Use of antidepressants was significantly associated with the response rate (*p* < .001). This is not remarkable, because the use of antidepressant within the control group means that these patients received an active treatment, while many of those not receiving antidepressants did not get an active treatment. After excluding waitlist control groups, the association between the use of antidepressants and the response rate within the control conditions was still significant (*p* = .002), but not after adding the study characteristics as predictors, including (*p* = 1.00) or excluding baseline severity (*p* = .93).

## Discussion

We examined in a large meta-analytic dataset of psychological treatments of depression if the proportion of users of antidepressants was associated with the effects found for these treatments. We found few indications that antidepressant use was associated with the effects of the treatments. We found that antidepressant use was associated with higher response rates within the control conditions, but not with the relative effects of the treatments compared to the control groups.

We indicated that there are three possible association between the use of antidepressants and psychotherapy: there is no association, the use of antidepressants reduces the effects of psychotherapies, or the use of antidepressants increase the effects of psychotherapies. Our results suggest that the first possibility is probably correct and that the use of antidepressants is not interfering with the effects of psychological treatments. This is in line with our previous research from the few randomized trials that can assess the effects of therapy, antidepressants, and combined treatment at the same time [7].

This is good news from a clinical perspective. Apparently, patients can start with psychotherapy and do not have to be afraid that the effects of the therapy will be smaller when they use antidepressants. It has been well-established that combined treatment is more effective than either psychotherapy or pharmacotherapy alone [3], but patients may prefer to start with one treatment instead of starting with the combination right away. In such cases, it is good to know that the new treatment will also be as effective when started later and in combination with the other.

We did find that the response rates within the control conditions were associated with more use of antidepressants, although this was no longer significant after adjusting for the study characteristics. In principle, it makes sense that people who use antidepressants have a higher chance of responding because they get an active treatment (antidepressants), while in the control group. People in control conditions not using antidepressants often do not get any active treatment at all. It remains unclear, however, why we did not find the same in the response rates within the treatment conditions. It would have been expected that the response rates would also be higher when patients get combined treatment instead of only psychotherapy. This finding is not easy to explain and definitely needs further exploration.

This study has several strengths. One important strength is that the number of available studies is large, providing sufficient statistical power to examine predictors of outcome. We also included enough trials in many relevant populations and target groups, different types of psychotherapies, and across different countries and control conditions.

However, there are also several important limitations that have to be acknowledged. First, our data do not allow to examine specific types of antidepressants, because studies typically only report the general use of antidepressants, without specifics. The data also do not allow to check the quality of the pharmacotherapy. Furthermore, in most trials, participants were already using antidepressants before the start of the trials and were on a stable dose. That means that the finding of no interaction may not be valid when patients start the two treatments at the same time, or first start with psychotherapy and then antidepressants. We also limited the analyses to the short-term effects of the treatment, and not to longer-term follow-up outcomes, because of the large number of trials and the major effort this would require. Another limitation of our study was the large number of low-quality trials as well as the high levels of heterogeneity in most analyses. Lastly, all our analyses are based on study-level information. This can lead to so-called “aggregation bias,” where associations on a study level (i.e., that studies’ percentage of antidepressant use is not associated with their overall effect size) may differ or even reverse on the patient level [43]. To rule out such biases, separate effect size data for patients with and without antidepressant use would be needed from each study, but this is typically not reported. Individual participant data (IPD) meta-analyses may also be used in the future to determine if associations differ on the patient level.

Despite these limitations, however, this study supports the hypothesis that the use of antidepressants does not interfere with the effects of psychotherapy. And that both can be used simultaneously without the effects of psychotherapy being reduced by antidepressants.

## Supporting information

Cuijpers et al. supplementary material Cuijpers et al. supplementary material

## Supplementary material

The supplementary material for this article can be found at https://doi.org/10.1192/j.eurpsy.2023.2437.
