# Supplementary material for: Does the use of pharmacotherapy interact with the effects of psychotherapy? A meta-analytic review
Source: Eur Psychiatry. 2023 Aug 3;66(1):e63. doi: 10.1192/j.eurpsy.2023.2437 (PMC10594340; doi:10.1192/j.eurpsy.2023.2437)
Supplement: Cuijpers et al. supplementary material [file S0924933823024379sup001.docx]

# Appendices

[Appendix A. References of included studies 2](#_Toc134781497)

[Appendix B. Selected characteristics of included studies 17](#_Toc134781498)

[Appendix C. Regression models of antidepressant use in trials comparing psychotherapy with control groups with baseline severity included 23](#_Toc134781499)

[Appendix D. Regression models of antidepressant use in trials comparing psychotherapy with control groups: Zero versus all participants using antidepressants 24](#_Toc134781500)

# Appendix A. References of included studies

| Study | Full reference |
| --- | --- |
| Ahmadpanah, 2017a | [Ahmadpanah M, Nazaribadie M, Aghaei E, Ghaleiha A, Bakhtiari A, Haghighi M, et al. Influence of adjuvant detached mindfulness and stress management training compared to pharmacologic treatment in primiparae with postpartum depression. Archives of women's mental health [Internet]. 2017:[1-9 pp.].](http://onlinelibrary.wiley.com/o/cochrane/clcentral/articles/997/CN-01393997/frame.html) |
| Alexopoulos, 2016 | Alexopoulos GS, Raue PJ, McCulloch C, Kanellopoulos D, Seirup JK, Sirey JA, et al. Clinical case management versus case management with problem-solving therapy in low-income, disabled elders with major depression: A randomized clinical trial. American Journal of Geriatric Psychiatry. 2016;24(1):50-9. |
| Alhusen, 2021 | Alhusen, J. L., et al. (2021). "A pilot study of a group-based perinatal depression intervention on reducing depressive symptoms and improving maternal-fetal attachment and maternal sensitivity." Arch Womens Ment Health 24(1): 145-154. |
| Allart van Dam, 2003 | Allart-Van Dam E, Hosman CMH, Hoogduin CAL, Schaap CPDR. The coping with depression course: Short-term outcomes and mediating effects of a randomized controlled trial in the treatment of subclinical depression. Behavior Therapy. 2003;34(3):381-96. |
| Ammerman, 2013 | Ammerman RT, Putnam FW, Altaye M, Stevens J, Teeters AR, Van Ginkel JB. A clinical trial of in-home CBT for depressed mothers in home visitation. Behavior Therapy. 2013;44(3):359-72. |
| Andersson, 2005 | Andersson G, Bergström J, Holländare F, Carlbring P, Kaldo V, Ekselius L. Internet-based self-help for depression: Randomised controlled trial. British Journal of Psychiatry. 2005;187(5):456-61. |
| Arean, 1993 | Arean PA, Perri MG, Nezu AM, Schein RL, Christopher F, Joseph TX. Comparative effectiveness of social problem-solving therapy and reminiscence therapy as treatments for depression in older adults. Journal of Consulting and Clinical Psychology. 1993;61(6):1003-10. |
| Arjadi, 2018 | Arjadi R, Nauta MH, Scholte WF, et al. Internet-based behavioural activation with lay counsellor support versus online minimal psychoeducation without support for treatment of depression: a randomised controlled trial in Indonesia. The lancet psychiatry 2018; 5(9): 707‐16. |
| Ashouri, 2013 | Ashouri A, Atef-Vahid MK, Gharaee B, Rasoulian M. Effectiveness of meta-cognitive and cognitive-behavioral therapy in patients with major depressive disorder. Iranian Journal of Psychiatry and Behavioral Sciences. 2013;7(2):24-34. |
| Au, 2022 | Au, A., Nan, H., Sum, R., Ng, F., Kwong, A., & Wong, S. (2022). Cognitive behavioural therapy for adherence and sub-clinical depression in type 2 diabetes: a randomised controlled trial (abridged secondary publication). Hong Kong Med J, 28 Suppl 3(3), 21-23. |
| Ayen, 2004 | Ayen I, Hautzinger M. Cognitive behavior therapy for depression in menopausal women. A controlled, randomized treatment study. Zeitschrift fur Klinische Psychologie und Psychotherapie. 2004;33(4):290-9. |
| Barber, 2012 | Barber JP, Barrett MS, Gallop R, Rynn MA, Rickels K. Short-term dynamic psychotherapy versus pharmacotherapy for major depressive disorder: A randomized, placebo-controlled trial. Journal of Clinical Psychiatry. 2012;73(1):66-73. |
| Barnhofer, 2009 | Barnhofer T, Crane C, Hargus E, Amarasinghe M, Winder R, Williams JM. Mindfulness-based cognitive therapy as a treatment for chronic depression: A preliminary study. Behaviour Research and Therapy. 2009;47(5):366-73. |
| Barrett, 2001 | Barrett JE, Williams Jr JW, Oxman TE, Frank E, Katon W, Sullivan M, et al. Treatment of dysthymia and minor depression in primary care: A ramdomized trial in patients aged 18 to 59 years. Journal of Family Practice. 2001;50(5):405-12. |
| Bastos, 2015 | Bastos AG, Guimaraes LSP, Trentini CM. The efficacy of long-term psychodynamic psychotherapy, fluoxetine and their combination in the outpatient treatment of depression. Psychotherapy Research. 2015;25(5):612-624. |
| Baumeister, 2021 | Baumeister, H., et al. (2021). "Effectiveness of a Guided Internet- and Mobile-Based Intervention for Patients with Chronic Back Pain and Depression (WARD-BP): A Multicenter, Pragmatic Randomized Controlled Trial." Psychchosom 90(4): 255-268. |
| Baumgartner, 2021 | Baumgartner, C., et al. (2021). ""Take Care of You" - Efficacy of integrated, minimal-guidance, internet-based self-help for reducing co-occurring alcohol misuse and depression symptoms in adults: Results of a three-arm randomized controlled trial." Drug Alcohol Depend 225: 108806. |
| Beach, 1992 | Beach SR, O'Leary KD. Treating depression in the context of marital discord: Outcome and predictors of response of marital therapy versus cognitive therapy. Behavior Therapy. 1992;23(4):507-28. |
| Bedard, 2014 | Bedard M, Felteau M, Marshall S, Cullen N, Gibbons C, Dubois S, et al. Mindfulness-based cognitive therapy reduces symptoms of depression in people with a traumatic brain injury: Results from a randomized controlled trial. Journal of Head Trauma Rehabilitation. 2014;29(4):E13-E22. |
| Beeber, 2010 | Beeber LS, Holditch-Davis D, Perreira K, Schwartz TA, Lewis V, Blanchard H, et al. Short-term in-home intervention reduces depressive symptoms in Early Head Start Latina mothers of infants and toddlers. Research in Nursing and Health. 2010;33(1):60-76. |
| Bellack, 1981 | Bellack AS, Hersen M, Himmelhoch J. Social skills training compared with pharmacotherapy and psychotherapy in the treatment of unipolar depression. American Journal of Psychiatry. 1981;138(12):1562-7. |
| Bellino, 2006 | Bellino S, Zizza M, Rinaldi C, Bogetto F. Combined treatment of major depression in patients with borderline personality disorder: A comparison with pharmacotherapy. Canadian Journal of Psychiatry. 2006;51(7):453-60. |
| Bendig, 2021 | Bendig, E., et al. (2021). "Lessons learned from an attempted randomized-controlled feasibility trial on “WIDeCAD” - An internet-based depression treatment for people living with coronary artery disease (CAD)." Internet Interventions 24. |
| Berger, 2011 | Berger T, Hämmerli K, Gubser N, Andersson G, Caspar F. Internet-based treatment of depression: A randomized controlled trial comparing guided with unguided self-help. Cognitive Behaviour Therapy. 2011;40(4):251-66. |
| Berman, 2022 | Berman, M. I., Park, J., Kragenbrink, M. E., & Hegel, M. T. (2022). Accept Yourself! A Pilot Randomized Controlled Trial of a Self-Acceptance-Based Treatment for Large-Bodied Women With Depression. Behav Ther, 53(5), 913-926. doi:10.1016/j.beth.2022.03.002 |
| Beutel, 2014 | Beutel ME, Weissflog G, Leuteritz K, Wiltink J, Haselbacher A, Ruckes C, et al. Efficacy of short-term psychodynamic psychotherapy (STPP) with depressed breast cancer patients: Results of a randomized controlled multicenter trial. Annals of Oncology. 2014;25(2):378-84. |
| Blackburn, 1981 | Blackburn IM, Bishop S, Glen AIM. The efficacy of cognitive therapy in depression: A treatment trial using cognitive therapy and pharmacotherapy, each alone and in combination. British Journal of Psychiatry. 1981;139(3):181-9. |
| Blom, 2007 | Blom MB, Jonker K, Dusseldorp E, Spinhoven P, Hoencamp E, Haffmans J, et al. Combination treatment for acute depression is superior only when psychotherapy is added to medication. Psychotherapy and Psychosomatics. 2007;76(5):289-97. |
| Boele, 2018 | Boele FW, Klein M, Verdonck-de Leeuw IM, et al. Internet-based guided self-help for glioma patients with depressive symptoms: a randomized controlled trial. Journal of neuro-oncology 2018; 137(1): 191‐203. |
| Boeschoten, 2017 | Boeschoten, R. E., Dekker, J., Uitdehaag, B. M. J., Beekman, A. T. F., Hoogendoorn, A. W., Collette, E. H., . . . Van Oppen, P. (2017). Internet-based treatment for depression in multiple sclerosis: A randomized controlled trial. Multiple Sclerosis, 23(8), 1112-1122. |
| Bowman, 1995 | Bowman D, Scogin F, Lyrene B. The efficacy of self-examination therapy and cognitive bibliotherapy in the treatment of mild to moderate depression. Psychotherapy Research. 1995;5(2):131-40. |
| Browne, 2002 | Browne G, Steiner M, Roberts J, Gafni A, Byrne C, Dunn E, et al. Sertraline and/or interpersonal psychotherapy for patients with dysthymic disorder in primary care: 6-month comparison with longitudinal 2-year follow-up of effectiveness and costs. Journal of Affective Disorders. 2002;68(2-3):317-30. |
| Buntrock, 2015 | Buntrock C, Ebert D, Lehr D, Riper H, Smit F, Cuijpers P, et al. Effectiveness of a web-based cognitive behavioural intervention for subthreshold depression: Pragmatic randomised controlled trial. Psychotherapy and Psychosomatics. 2015;84(6):348-58. |
| Burnand, 2002 | Burnand Y, Andreoli A, Kolatte E, Venturini A, Rosset N. Psychodynamic psychotherapy and clomipramine in the treatment of major depression. Psychiatric Services. 2002;53(5):585-90. |
| Burns, 2013 | Burns A, O'Mahen H, Baxter H, Bennert K, Wiles N, Ramchandani P, et al. A pilot randomised controlled trial of cognitive behavioural therapy for antenatal depression. BMC psychiatry. 2013;13:33. |
| Carr, 2017 | Carr, A., Finnegan, L., Griffin, E., Cotter, P., & Hyland, A. (2017). A Randomized Controlled Trial of the Say Yes to Life (SYTL) Positive Psychology Group Psychotherapy Program for Depression: An Interim Report. Journal of Contemporary Psychotherapy, 47(3), 153-161. |
| Carta, 2012 | Carta MG, Petretto D, Adamo S, Bhat KM, Lecca ME, Mura G, et al. Counseling in primary care improves depression and quality of life. Clinical Practice and Epidemiology in Mental Health. 2012;8. |
| Casanas, 2012 | Casanas R, Catalan R, del Val JL, Real J, Valero S, Casas M. Effectiveness of a psycho-educational group program for major depression in primary care: A randomized controlled trial. BMC psychiatry. 2012;12:230. |
| Chan, 2012 | Chan AS, Wong QY, Sze SL, Kwong PP, Han YM, Cheung MC. A Chinese Chan-based mind-body intervention for patients with depression. Journal of Affective Disorders. 2012;142(1-3):283-9. |
| Chesney, 2003 | Chesney MA, Chambers DB, Taylor JM, Johnson LM, Folkman S. Coping effectiveness training for men living with HIV: Results from a randomized clinical trial testing a group-based intervention. Psychosomatic Medicine. 2003;65(6):1038-46. |
| Chetty, 2013 | Chetty D, Hoque ME. Effectiveness of a nurse facilitated cognitive group intervention among mild to moderately-depressed-women in KwaZulu-Natal, South Africa. African journal of psychiatry. 2013;16(1):29-34. |
| Chiang, 2015 | Chiang KJ, Chen TH, Hsieh HT, Tsai JC, Ou KL, Chou KR. One-year follow-up of the effectiveness of cognitive behavioral group therapy for patients' depression: A randomized, single-blinded, controlled study. Scientific World Journal. 2015;2015:Article ID 373149. |
| Choi, 2020 | Choi H, Jung DJ, Jeon YH, Kim MJ. The effects of combining art psychotherapy with pharmacotherapy in treating major depressive disorder: randomized control study. Arts in psychotherapy. 2020;70. |
| Choi, 2020b | Choi NG, Marti CN, Wilson NL, et al. Effect of Telehealth Treatment by Lay Counselors vs by Clinicians on Depressive Symptoms Among Older Adults Who Are Homebound: A Randomized Clinical Trial. JAMA Netw Open. 2020;3(8):e2015648. |
| Choy, 2016 | Choy JC, Lou VW. Effectiveness of the modified instrumental reminiscence intervention on psychological well-being among community-dwelling chinese older adults: A randomized controlled trial. American Journal of Geriatric Psychiatry. 2016;24(1):60-9. |
| Cohen, 2010 | Cohen S, O'Leary KD, Foran H. A randomized clinical trial of a brief, problem-focused couple therapy for depression. Behavior Therapy. 2010;41(4):433-46. |
| Corruble, 2016 | Corruble E, Swartz HA, Bottai T, Vaiva G, Bayle F, Llorca PM, et al. Telephone-administered psychotherapy in combination with antidepressant medication for the acute treatment of major depressive disorder. Journal of Affective Disorders. 2016;190:6-11. |
| Cramer, 2011 | Cramer H, Salisbury C, Conrad J, Eldred J, Araya R. Group cognitive behavioural therapy for women with depression: Pilot and feasibility study for a randomised controlled trial using mixed methods. BMC psychiatry. 2011;11:82. |
| De Jong, 2018 | De Jong M, Peeters F, Gard T, et al. A randomized controlled pilot study on mindfulness-based cognitive therapy for unipolar depression in patients with chronic pain. Journal of clinical psychiatry 2018; 79(1): 26‐34. |
| De Jonghe, 2001 | de Jonghe F, Kool S, van Aalst G, Dekker J, Peen J. Combining psychotherapy and antidepressants in the treatment of depression. Journal of Affective Disorders. 2001;64(2-3):217-29. |
| De Mello, 2001 | de Mello MF, Myczcowisk LM, Menezes PR. A randomized controlled trial comparing moclobemide and moclobemide plus interpersonal psychotherapy in the treatment of dysthymic disorder. Journal of Psychotherapy Practice and Research. 2001;10(2):117-23. |
| Dekker, 2012 | Dekker RL, Moser DK, Peden AR, Lennie TA. Cognitive therapy improves three-month outcomes in hospitalized patients with heart failure. Journal of cardiac failure. 2012;18(1):10-20. |
| Dennis, 2020 | Dennis CL, Grigoriadis S, Zupancic J, Kiss A, Ravitz P. Telephone-based nurse-delivered interpersonal psychotherapy for postpartum depression: nationwide randomised controlled trial. Br J Psychiatry. 2020;216(4):189-196. |
| Denton, 2012 | Denton WH, Wittenborn AK, Golden RN. Augmenting antidepressant medication treatment of depressed women with emotionally focused therapy for couples: A randomized pilot study. Journal of marital and family therapy. 2012;38 Suppl 1:23-38. |
| DeRubeis, 2005 | DeRubeis RJ, Hollon SD, Amsterdam JD, Shelton RC, Young PR, Salomon RM, et al. Cognitive therapy vs medications in the treatment of moderate to severe depression. Archives of General Psychiatry. 2005;62(4):409-16. |
| Desautels, 2018 | Desautels C, Savard J, Ivers H, Savard MH, Caplette-Gingras A. Treatment of depressive symptoms in patients with breast cancer: a randomized controlled trial comparing cognitive therapy and bright light therapy. Health psychology 2018; 37(1): 1‐13. |
| Dimidjian, 2006 | Dimidjian S, Hollon SD, Dobson KS, Schmaling KB, Kohlenberg RJ, Addis ME, et al. Randomized trial of behavioral activation, cognitive therapy, and antidepressant medication in the acute treatment of adults with major depression. Journal of Consulting and Clinical Psychology. 2006;74(4):658-70. |
| Dimidjian, 2017 | Dimidjian, S., Goodman, S., Sherwood, N., Simon, G., Ludman, E., Gallop, R., . . . Beck, A. (2017). A Pragmatic Randomized Clinical Trial of Behavioral Activation for Depressed Pregnant Women. Journal of Consulting and Clinical Psychology, 85(1), 26-36 |
| Dindo, 2012 | Dindo L, Recober A, Marchman JN, Turvey C, O'Hara MW. One-day behavioral treatment for patients with comorbid depression and migraine: A pilot study. Behaviour Research and Therapy. 2012;50(9):537-43. |
| Dindo, 2020 | Dindo, L. N., Recober, A., Calarge, C. A., Zimmerman, B. M., Weinrib, A., Marchman, J. N., & Turvey, C. (2019). One-Day Acceptance and Commitment Therapy Compared to Support for Depressed Migraine Patients: a Randomized Clinical Trial. Neurotherapeutics. doi:10.1007/s13311-019-00818-0 |
| Dobkin, 2011 | Dobkin RD, Menza M, Allen LA, Gara MA, Mark MH, Tiu J, et al. Cognitive-behavioral therapy for depression in Parkinson's disease: A randomized, controlled trial. American Journal of Psychiatry. 2011;168(10):1066-74. |
| Dobkin, 2020 | Dobkin RD, Mann SL, Gara MA, Interian A, Rodriguez KM, Menza M. Telephone-based cognitive behavioral therapy for depression in Parkinson disease: A randomized controlled trial. Neurology. 2020;94(16):e1764-e1773. |
| Dobkin, 2021 | Dobkin, R. D., et al. (2021). "Innovating Parkinson's Care: A Randomized Controlled Trial of Telemedicine Depression Treatment." Movement Disorders 36(11): 2549-2558. |
| Doering, 2013 | Doering LV, Chen B, Cross Bodan R, Magsarili MC, Nyamathi A, Irwin MR. Early cognitive behavioral therapy for depression after cardiac surgery. Journal of Cardiovascular Nursing. 2013;28(4):370-9. |
| Dong, 2019 | Dong, X., Sun, G., Zhan, J., Liu, F., Ma, S., Li, P., . . . Liu, Y. (2019). Telephone-based reminiscence therapy for colorectal cancer patients undergoing postoperative chemotherapy complicated with depression: a three-arm randomised controlled trial. Supportive care in cancer : official journal of the Multinational Association of Supportive Care in Cancer, 27(8), 2761-2769. doi:10.1007/s00520-018-4566-6 |
| Dozois, 2009 | Dozois DJ, Bieling PJ, Patelis-Siotis I, Hoar L, Chudzik S, McCabe K, et al. Changes in self-schema structure in cognitive therapy for major depressive disorder: A randomized clinical trial. Journal of Consulting and Clinical Psychology. 2009;77(6):1078-88. |
| Duarte, 2009 | Duarte PS, Miyazaki MC, Blay SL, Sesso R. Cognitive-behavioral group therapy is an effective treatment for major depression in hemodialysis patients. Kidney international. 2009;76(4):414-21. |
| Dwight-Johnson, 2011 | Dwight-Johnson M, Aisenberg E, Golinelli D, Hong S, O'Brien M, Ludman E. Telephone-based cognitive-behavioral therapy for Latino patients living in rural areas: A randomized pilot study. Psychiatric Services. 2011;62(8):936-42. |
| Ebert, 2018 | Ebert DD, Buntrock C, Lehr D, et al. Effectiveness of Web- and Mobile-Based Treatment of Subthreshold Depression With Adherence-Focused Guidance: a Single-Blind Randomized Controlled Trial. Behavior therapy 2018; 49(1): 71‐83. |
| Ekers, 2011 | Ekers D, Richards D, McMillan D, Bland JM, Gilbody S. Behavioural activation delivered by the non-specialist: Phase II randomised controlled trial. The British Journal of Psychiatry. 2011;198(1):66-72. |
| Ekkers, 2011 | Ekkers W, Korrelboom K, Huijbrechts I, Smits N, Cuijpers P, Gaag M. Competitive memory training for treating depression and rumination in depressed older adults: A randomized controlled trial. Behaviour Research and Therapy. 2011;49(10):588-96. |
| Elkin, 1989 | Elkin I, Shea MT, Watkins JT, Imber SD, Sotsky SM, Collins JF, et al. National institute of mental health treatment of depression collaborative research program. General effectiveness of treatments. Archives of General Psychiatry. 1989;46(11):971-82; discussion 83. |
| Euteneuer, 2017 | Euteneuer, F., Dannehl, K., Del Rey, A., Engler, H., Schedlowski, M., & Rief, W. (2017). Immunological effects of behavioral activation with exercise in major depression: An exploratory randomized controlled trial. Translational Psychiatry, 7(5). |
| Euteneuer, 2022 | Euteneuer, F., Neuert, M., Salzmann, S., Fischer, S., Ehlert, U., & Rief, W. (2022). Does psychological treatment of major depression reduce cardiac risk biomarkers? An exploratory randomized controlled trial. Psychological medicine, 1-15. doi:10.1017/S0033291722000447 |
| Fann, 2015 | Fann JR, Bombardier CH, Vannoy S, Dyer J, Ludman E, Dikmen S, et al. Telephone and in-person cognitive behavioral therapy for major depression after traumatic brain injury: A randomized controlled trial. Journal of neurotrauma. 2015;32(1):45-57. |
| Finkenzeller, 2009 | Finkenzeller W, Zobel I, Rietz S, Schramm E, Berger M. [Interpersonal psychotherapy and pharmacotherapy for post-stroke depression. Feasibility and effectiveness]. Der Nervenarzt. 2009;80(7):805-12. |
| Fledderus, 2012 | Fledderus M, Bohlmeijer ET, Pieterse ME, Schreurs KM. Acceptance and commitment therapy as guided self-help for psychological distress and positive mental health: A randomized controlled trial. Psychological Medicine. 2012;42(3):485-95. |
| Floyd, 2004 | Floyd M, Scogin F, McKendree-Smith NL, Floyd DL, Rokke PD. Cognitive therapy for depression: A comparison of individual psychotherapy and bibliotherapy for depressed older adults. Behavior modification. 2004;28(2):297-318. |
| Folke, 2012 | Folke F, Parling T, Melin L. Acceptance and commitment therapy for depression: A preliminary randomized clinical trial for unemployed on long-term sick leave. Cognitive and Behavioral Practice. 2012;19(4):583-94. |
| Fonagy, 2015 | Fonagy P, Rost F, Carlyle JA, McPherson S, Thomas R, Pasco Fearon RM, et al. Pragmatic randomized controlled trial of long-term psychoanalytic psychotherapy for treatment-resistant depression: The Tavistock Adult Depression Study (TADS). World Psychiatry. 2015;14(3):312-21. |
| Forand, 2018 | Forand NR, Barnett JG, Strunk DR, Hindiyeh MU, Feinberg JE, Keefe JR. Efficacy of Guided iCBT for Depression and Mediation of Change by Cognitive Skill Acquisition. Behavior Therapy 2018; 49(2): 295-307. |
| Forsell, 2017 | Forsell E, Bendix M, Holländare F, et al. Internet delivered cognitive behavior therapy for antenatal depression: A randomised controlled trial. Journal of Affective Disorders 2017; 221: 56-64. |
| Freedland, 2009 | Freedland KE, Skala JA, Carney RM, Rubin EH, Lustman PJ, D·vila-Rom·n VG, et al. Treatment of depression after coronary artery bypass surgery: A randomized controlled trial. Archives of General Psychiatry. 2009;66(4):387-96. |
| Freedland, 2015 | Freedland KE, Carney RM, Rich MW, Steinmeyer BC, Rubin EH. Cognitive behavior therapy for depression and self-care in heart failure patients: A randomized clinical trial. JAMA Internal Medicine. 2015;175(11):1773-82. |
| Funderburk, 2021 | Funderburk, J. S., et al. (2021). "Treating depressive symptoms among veterans in primary care: A multi-site RCT of brief behavioral activation." J Affect Disord 283: 11-19. |
| Gater, 2010 | Gater R, Waheed W, Husain N, Tomenson B, Aseem S, Creed F. Social intervention for British Pakistani women with depression: Randomised controlled trial. The British Journal of Psychiatry. 2010;197(3):227-33. |
| Gaudiano, 2015 | Gaudiano BA, Busch AM, Wenze SJ, Nowlan K, Epstein-Lubow G, Miller IW. Acceptance-based behavior therapy for depression with psychosis: Results from a pilot feasibility randomized controlled trial. Journal of Psychiatric Practice. 2015;21(5):320-33. |
| Gawrysiak, 2009 | Gawrysiak M, Nicholas C, Hopko DR. Behavioral activation for moderately depressed university students: Randomized controlled trial. Journal of Counseling Psychology. 2009;56(3):468-75. |
| Gellis, 2008 | Gellis ZD, McGinty J, Tierney L, Jordan C, Burton J, Misener E. Randomized controlled trial of problem-solving therapy for minor depression in home care. Research on Social Work Practice. 2008;18(6):596-606. |
| Gellis, 2010 | Gellis ZD, Bruce ML. Problem solving therapy for subthreshold depression in home healthcare patients with cardiovascular disease. The American Journal of Geriatric Psychiatry. 2010;18(6):464-74. |
| Geraedts, 2014 | Geraedts AS, Kleiboer AM, Wiezer NM, van Mechelen W, Cuijpers P. Short-term effects of a web-based guided self-help intervention for employees with depressive symptoms: Randomized controlled trial. Journal of Medical Internet Research. 2014;16(5):e121. |
| Ghorbani, 2021 | Ghorbani, V., et al. (2021). "Efficacy of acceptance and commitment therapy (ACT) on depression, pain acceptance, and psychological flexibility in married women with breast cancer: a pre- and post-test clinical trial." Trends Psychiatry Psychother 43(2): 126-133. |
| Gitlin, 2013 | Gitlin LN, Harris LF, McCoy MC, Chernett NL, Pizzi LT, Jutkowitz E, et al. A home-based intervention to reduce depressive symptoms and improve quality of life in older African Americans: A randomized trial. Annals of Internal Medicine. 2013;159(4):243-52. |
| Goodman, 2015 | Goodman JH, Prager J, Goldstein R, Freeman M. Perinatal Dyadic Psychotherapy for postpartum depression: A randomized controlled pilot trial. Archives of Women's Mental Health. 2015;18(3):493-506. |
| Grote, 2009 | Grote NK, Swartz HA, Geibel SL, Zuckoff A, Houck PR, Frank E. A randomized controlled trial of culturally relevant, brief interpersonal psychotherapy for perinatal depression. Psychiatric Services. 2009;60(3):313-21. |
| Guo, 2022 | Guo, H., Ren, Y., Huang, B., Wang, J., Yang, X., & Wang, Y. (2022). Psychological Status, Compliance, Serum Brain-Derived Neurotrophic Factor, and Nerve Growth Factor Levels of Patients with Depression after Augmented Mindfulness-Based Cognitive Therapy. Genet Res (Camb), 2022, 1097982. doi:10.1155/2022/1097982 |
| Hagen, 2017 | Hagen, R., Hjemdal, O., Solem, S., Kennair, L. E. O., Nordahl, H. M., Fisher, P., & Wells, A. (2017). Metacognitive therapy for depression in adults: A waiting list randomized controlled trial with six months follow-up. Frontiers in Psychology, 8. |
| Hallford, 2016 | Hallford DJ, Mellor D. Autobiographical memory-based intervention for depressive symptoms in young adults: A randomized controlled trial of cognitive-reminiscence therapy. Psychotherapy and Psychosomatics. 2016;85(4):246-9. |
| Hallgren, 2015 | Hallgren M, Kraepelien M, Öjehagen A, Lindefors N, Zeebari Z, Kaldo V, et al. Physical exercise and internet-based cognitive-behavioural therapy in the treatment of depression: Randomised controlled trial. British Journal of Psychiatry. 2015;207(3):227-34. |
| Hamamci, 2006 | Hamamci Z. Integrating psychodrama and cognitive behavioral therapy to treat moderate depression. Arts in Psychotherapy. 2006;33(3):199-207. |
| Haringsma, 2006 | Haringsma R, Engels G, Cuijpers P, Spinhoven P. Effectiveness of the Coping With Depression (CWD) course for older adults provided by the community-based mental health care system in the Netherlands: A randomized controlled field trial. International Psychogeriatrics. 2006;18(02):307-25. |
| Harley, 2008 | Harley R, Sprich S, Safren S, Jacobo M, Fava M. Adaptation of dialectical behavior therapy skills training group for treatment-resistant depression. Journal of Nervous and Mental Disease. 2008;196(2):136-43. |
| Hautzinger, 1996 | Hautzinger M, De Jong-Meyer R, Treiber R, Rudolf GAE, Thien U. Efficacy of cognitive behavior therapy, pharmacotherapy, and the combination of both in non-melancholic, unipolar depression. Zeitschrift fur Klinische Psychologie. 1996;25(2):130-45. |
| Hautzinger, 2004 | Hautzinger M, Welz S. Kognitive Verhaltenstherapie bei Depressionen im Alter: Ergebnisse einer kontrollierten Vergleichsstudie unter ambulanten Bedingungen an Depressionen mittleren Schweregrads. = Cognitive behavioral therapy for depressed older outpatients: A controlled, randomized trial. Zeitschrift für Gerontologie und Geriatrie. 2004;37(6):427-35. |
| Hayman, 1980 | Hayman PM, Cope CS. Effects of assertion training on depression. Journal of clinical psychology. 1980;36(2):534-43. |
| He, 2019 | He, H.-L., Zhang, M., Gu, C.-Z., Xue, R.-R., Liu, H.-X., Gao, C.-F., & Duan, H.-F. (2019). Effect of Cognitive Behavioral Therapy on Improving the Cognitive Function in Major and Minor Depression. The Journal of nervous and mental disease, 207(4), 232-238. doi:10.1097/NMD.0000000000000954 |
| Heckman, 2011 | Heckman TG, Sikkema KJ, Hansen N, Kochman A, Heh V, Neufeld S, et al. A randomized clinical trial of a coping improvement group intervention for HIV-infected older adults. Journal of behavioral medicine. 2011;34(2):102-11. |
| Heckman, 2017 | Heckman, T. G., Heckman, B. D., Anderson, T., Lovejoy, T. I., Markowitz, J. C., Shen, Y., & Sutton, M. (2017). Tele-interpersonal psychotherapy acutely reduces depressive symptoms in depressed HIV-infected rural persons: A randomized clinical trial. Behavioral Medicine, 43(4), 285-295. |
| Hegerl, 2010 | Hegerl U, Hautzinger M, Mergl R, Kohnen R, Sch¸tze M, Scheunemann W, et al. Effects of pharmacotherapy and psychotherapy in depressed primary-care patients: A randomized, controlled trial including a patients' choice arm. International Journal of Neuropsychopharmacology. 2010;13(1):31-44. |
| Hellerstein, 2001 | Hellerstein DJ, Little SA, Samstag LW, Batchelder S, Muran JC, Fedak M, et al. Adding group psychotherapy to medication treatment in dysthymia: A randomized prospective pilot study. Journal of Psychotherapy Practice and Research. 2001;10(2):93-103. |
| Hemanny, 2019 | Hemanny, C., Carvalho, C., Maia, N., Reis, D., Botelho, A. C., Bonavides, D., . . . De Oliveira, I. R. (2019). Efficacy of trial-based cognitive therapy, behavioral activation and treatment as usual in the treatment of major depressive disorder: Preliminary findings from a randomized clinical trial. CNS Spectrums. doi:10.1017/S1092852919001457 |
| Hermanns, 2015 | Hermanns N, Schmitt A, Gahr A, Herder C, Nowotny B, Roden M, et al. The effect of a diabetes-specific cognitive behavioral treatment program (DIAMOS) for patients with diabetes and subclinical depression: Results of a randomized controlled trial. Diabetes care. 2015;38(4):551-60. |
| Herrmann-Lingen, 2016 | Herrmann-Lingen C, Beutel ME, Bosbach A, Deter HC, Fritzsche K, Hellmich M, et al. A stepwise psychotherapy intervention for reducing risk in coronary artery disease (SPIRR-CAD): Results of an observer-blinded, multicenter, randomized trial in depressed patients with coronary artery disease. Psychosomatic Medicine. 2016;78(6):704-15. |
| Hoifodt, 2013 | Hoifodt, R. S., Lillevoll, K. R., Griffiths, K. M., Wilsgaard, T., Eisemann, M., Waterloo, K., & Kolstrup, N. (2013). The clinical effectiveness of web-based cognitive behavioral therapy with face-to-face therapist support for depressed primary care patients: randomized controlled trial. J Med Internet Res, 15(8), e153. doi:10.2196/jmir.2714 |
| Hollon, 1992 | Hollon SD, DeRubeis RJ, Evans MD, Wiemer MJ, Garvey MJ, Grove WM, et al. Cognitive therapy and pharmacotherapy for depression. Singly and in combination. Archives of General Psychiatry. 1992;49(10):774-81. |
| Hollon, 2014 | Hollon SD, DeRubeis RJ, Fawcett J, Amsterdam JD, Shelton RC, Zajecka J, et al. Effect of cognitive therapy with antidepressant medications vs antidepressants alone on the rate of recovery in major depressive disorder: A randomized clinical trial. JAMA psychiatry. 2014;71(10):1157-64. |
| Hsiao, 2011 | Hsiao FH, Jow GM, Lai YM, Chen YT, Wang KC, Ng SM, et al. The long-term effects of psychotherapy added to pharmacotherapy on morning to evening diurnal cortisol patterns in outpatients with major depression. Psychotherapy and Psychosomatics. 2011;80(3):166-72. |
| Hsiao, 2014 | Hsiao F-H, Lai Y-M, Chen Y-T, Yang T-T, Liao S-C, Ho RT, et al. Efficacy of psychotherapy on diurnal cortisol patterns and suicidal ideation in adjustment disorder with depressed mood. General Hospital Psychiatry. 2014;36(2):214-9. |
| Hummel, 2017 | Hummel, J., Weisbrod, C., Boesch, L., Himpler, K., Hauer, K., Hautzinger, M., . . . Kopf, D. (2017). AIDE–Acute Illness and Depression in Elderly Patients. Cognitive Behavioral Group Psychotherapy in Geriatric Patients With Comorbid Depression: A Randomized, Controlled Trial. Journal of the american medical directors association, 18(4), 341-349. |
| Jarrett, 1999 | Jarrett RB, Schaffer M, McIntire D, Witt-Browder A, Kraft D, Risser RC. Treatment of atypical depression with cognitive therapy or phenelzine: A double-blind, placebo-controlled trial. Archives of General Psychiatry. 1999;56(5):431-7. |
| Jelinek, 2016 | Jelinek L, Hauschildt M, Wittekind CE, Schneider BC, Kriston L, Moritz S. Efficacy of metacognitive training for depression: A randomized controlled trial. Psychotherapy and Psychosomatics. 2016;85(4):231-4. |
| Johnson, 2012 | Johnson JE, Zlotnick C. Pilot study of treatment for major depression among women prisoners with substance use disorder. Journal of Psychiatric Research. 2012;46(9):1174-83. |
| Johnson, 2019 | Johnson, J. E., Stout, R. L., Miller, T. R., Zlotnick, C., Cerbo, L. A., Andrade, J. T., . . . Wiltsey-Stirman, S. (2019). Randomized cost-effectiveness trial of group interpersonal psychotherapy (IPT) for prisoners with major depression. Journal of consulting and clinical psychology, 87(4), 392-406. doi:10.1037/ccp0000379 |
| Kamga, 2017 | Kamga, H., McCusker, J., Yaffe, M., Sewitch, M., Sussman, T., Strumpf, E., . . . Freeman, E. (2017). Self-care tools to treat depressive symptoms in patients with age-related eye disease: a randomized controlled clinical trial. Clinical & experimental ophthalmology, 45(4), 371-378. |
| Keeley, 2016 | Keeley RD, Brody DS, Engel M, Burke BL, Nordstrom K, Moralez E, et al. Motivational interviewing improves depression outcome in primary care: A cluster randomized trial. Journal of Consulting and Clinical Psychology. 2016;84(11):993-1007. |
| Keller, 2000 | Keller MB, McCullough JP, Klein DN, Arnow B, Dunner DL, Gelenberg AJ, et al. A comparison of nefazodone, the cognitive behavioral-analysis system of psychotherapy, and their combination for the treatment of chronic depression. The New England journal of medicine. 2000;342(20):1462-70. |
| Kenter, 2016 | Kenter, R. M. F., Cuijpers, P., Beekman, A., & van Straten, A. (2016). Effectiveness of a Web-based guided self-help intervention for outpatients with a depressive disorder: Short-term results from a randomized controlled trial. Journal of medical Internet research, 18(3). |
| Kessler, 2009 | Kessler D, Lewis G, Kaur S, Wiles N, King M, Weich S, et al. Therapist-delivered Internet psychotherapy for depression in primary care: A randomised controlled trial. Lancet. 2009;374(9690):628-34. |
| Khoshbooii, 2021 | Khoshbooii, R., et al. (2021). "Effects of Group and Individual Culturally Adapted Cognitive Behavioral Therapy on Depression and Sexual Satisfaction among Perimenopausal Women." Int J Environ Res Public Health 18(14). |
| Kim, 2018 | Kim YH, Choi KS, Han K, Kim HW. A psychological intervention programme for patients with breast cancer under chemotherapy and at a high risk of depression: a randomised clinical trial. Journal of clinical nursing 2018; 27(3‐4): 572‐81. |
| Kramer, 2021 | Krämer, L. V., et al. (2021). "Effectiveness of a Guided Web-Based Intervention to Reduce Depressive Symptoms before Outpatient Psychotherapy: A Pragmatic Randomized Controlled Trial." Psychosom 90(4): 233-242. |
| Laidlaw, 2008 | Laidlaw K, Davidson K, Toner H, Jackson G, Clark S, Law J, et al. A randomised controlled trial of cognitive behaviour therapy vs treatment as usual in the treatment of mild to moderate late life depression. International Journal of Geriatric Psychiatry. 2008;23(8):843-50. |
| Lam, 2013 | Lam RW, Parikh SV, Ramasubbu R, Michalak EE, Tam EM, Axler A, et al. Effects of combined pharmacotherapy and psychotherapy for improving work functioning in major depressive disorder. The British Journal of Psychiatry. 2013;203(5):358-65. |
| Lamers, 2010 | Lamers F, Jonkers CC, Bosma H, Kempen GI, Meijer JA, Penninx BW, et al. A minimal psychological intervention in chronically ill elderly patients with depression: A randomized trial. Psychotherapy and Psychosomatics. 2010;79(4):217-26. |
| Lappalainen, 2015 | Lappalainen P, Langrial S, Oinas-Kukkonen H, Tolvanen A, Lappalainen R. Web-based acceptance and commitment therapy for depressive symptoms with minimal support: A randomized controlled trial. Behavior modification. 2015;39(6):805-34. |
| Larcombe, 1984 | Larcombe NA, Wilson PH. An evaluation of cognitive-behaviour therapy for depression in patients with multiple sclerosis. The British Journal of Psychiatry. 1984;145:366-71. |
| Lee, 2021 | Lee, E., et al. (2021). "Community-Based Multi-Site Randomized Controlled Trial of Behavioral Activation for Patients with Depressive Disorders." Community mental health journal. |
| Lenze, 2020 | Lenze SN, Potts MA, Rodgers J, Luby J. Lessons learned from a pilot randomized controlled trial of dyadic interpersonal psychotherapy for perinatal depression in a low-income population. Journal of Affective Disorders. 2020;271:286-292. |
| Lerner, 2015 | Lerner D, Adler DA, Rogers WH, Chang H, Greenhill A, Cymerman E, et al. A randomized clinical trial of a telephone depression intervention to reduce employee presenteeism and absenteeism. Psychiatric Services. 2015;66(6):570-7. |
| Lesperance, 2007 | Lesperance F, Frasure-Smith N, Koszycki D, Laliberte MA, van Zyl LT, Baker B, et al. Effects of citalopram and interpersonal psychotherapy on depression in patients with coronary artery disease: The Canadian Cardiac Randomized Evaluation of Antidepressant and Psychotherapy Efficacy (CREATE) trial. JAMA. 2007;297(4):367-79. |
| Li, 2022 | Li, H., Yan, W., Wang, Q., Liu, L., Lin, X., Zhu, X., . . . Sun, X. (2022). Mindfulness-Based Cognitive Therapy Regulates Brain Connectivity in Patients With Late-Life Depression. Frontiers in Psychiatry, 13. doi:10.3389/fpsyt.2022.841461 |
| Linde, 2011 | Linde JA, Simon GE, Ludman EJ, Ichikawa LE, Operskalski BH, Arterburn D, et al. A randomized controlled trial of behavioral weight loss treatment versus combined weight loss/depression treatment among women with comorbid obesity and depression. Annals of Behavioral Medicine. 2011;41(1):119-30. |
| Liu, 2009 | Liu ET-H, Chen W-L, Li Y-H, Wang CH, Mok TJ, Huang HS. Exploring the efficacy of cognitive bibliotherapy and a potential mechanism of change in the treatment of depressive symptoms among the Chinese: A randomized controlled trial. Cognitive Therapy and Research. 2009;33(5):449-61. |
| Lloyd-Williams, 2018 | Lloyd-Williams M, Shiels C, Ellis J, et al. Pilot randomised controlled trial of focused narrative intervention for moderate to severe depression in palliative care patients: DISCERN trial. Palliative medicine 2018; 32(1): 206‐15. |
| Lovell, 2008 | Lovell K, Bower P, Richards D, Barkham Mi, Sibbald B, Roberts C, et al. Developing guided self-help for depression using the medical research council complex interventions framework: A description of the modelling phase and results of an exploratory randomised controlled trial. BMC psychiatry. 2008;8. |
| Lundgren, 2016 | Lundgren JG, Dahlstrom O, Andersson G, Jaarsma T, Karner Kohler A, Johansson P. The effect of guided web-based cognitive behavioral therapy on patients with depressive symptoms and heart failure: A pilot randomized controlled trial. Journal of Medical Internet Research. 2016;18(8):e194. |
| Lustman, 1998 | Lustman PJ, Griffith LS, Freedland KE, Kissel SS, Clouse RE. Cognitive behavior therapy for depression in type 2 diabetes mellitus. A randomized, controlled trial. Annals of Internal Medicine. 1998;129(8):613-21. |
| Lynch, 1997 | Lynch DJ, Tamburrino MB, Nagel R. Telephone counseling for patients with minor depression: Preliminary findings in a family practice setting. The Journal of family practice. 1997;44(3):293-8. |
| Lynch, 2003 | Lynch TR, Morse JQ, Mendelson T, Robins C. Dialectical behavior therapy for depressed older adults: A randomized pilot study. American Journal of Geriatric Psychiatry. 2003;11(1):33-45. |
| Macaskil, 1996 | Macaskill ND, Macaskill A. Rational-emotive therapy plus pharmacotherapy versus pharmacotherapy alone in the treatment of high cognitive dysfunction depression. Cognitive Therapy and Research. 1996;20(6):575-92. |
| MacPherson, 2013 | MacPherson H, Richmond S, Bland M, Brealey S, Gabe R, Hopton A, et al. Acupuncture and counselling for depression in primary care: A randomised controlled trial. PLoS medicine. 2013;10(9):e1001518. |
| Maina, 2005 | Maina G, Forner F, Bogetto F. Randomized controlled trial comparing brief dynamic and supportive therapy with waiting list condition in minor depressive disorders. Psychotherapy and Psychosomatics. 2005;74(1):43-50. |
| Maina, 2010 | Maina G, Rosso G, Rigardetto S, Chiado Piat S, Bogetto F. No effect of adding brief dynamic therapy to pharmacotherapy in the treatment of obsessive-compulsive disorder with concurrent major depression. Psychotherapy and Psychosomatics. 2010;79(5):295-302. |
| Maldonado, 1984 | Maldonado LA. Un modelo de terapia cognitiva desde la perspectiva de la psicología del aprendizaje. Anuario de psicología/The UB Journal of psychology. 1984(30):75-96. |
| Mansour, 2022 | Mansour, N., Labib, N., Khalil, M., & Esmat, S. (2022). Brief Cognitive Behavioral Therapy for Patients with Comorbid Depression and Type 2 Diabetes in an Urban Primary Care Facility: Randomized Controlled Trial. Open Access Macedonian Journal of Medical Sciences, 10, 60-67. doi:10.3889/oamjms.2022.7883 |
| Markowitz, 2005 | Markowitz JC, Kocsis JH, Bleiberg KL, Christos PJ, Sacks M. A comparative trial of psychotherapy and pharmacotherapy for "pure" dysthymic patients. Journal of Affective Disorders. 2005;89(1-3):167-75. |
| Matsuzaka, 2017 | Matsuzaka, C., Wainberg, M., Norcini, P. A., Hoffmann, E., Coimbra, B., Braga, R., . . . Mello, M. (2017). Task shifting interpersonal counseling for depression: a pragmatic randomized controlled trial in primary care. BMC Psychiatry, 17(1) |
| McClay, 2015 | McClay CA, Collins K, Matthews L, Haig C, McConnachie A, Morrison J, et al. A community-based pilot randomised controlled study of life skills classes for individuals with low mood and depression. BMC psychiatry. 2015;15:17. |
| McCusker, 2021 | McCusker, J., et al. (2021). "CanDirect: Effectiveness of a Telephone-Supported Depression Self-Care Intervention for Cancer Survivors." J Clin Oncol 39(10): 1150-1161. |
| McIndoo, 2016 | McIndoo CC, File AA, Preddy T, Clark CG, Hopko DR. Mindfulness-based therapy and behavioral activation: A randomized controlled trial with depressed college students. Behaviour Research and Therapy. 2016;77:118-28. |
| Mennen, 2021 | Mennen, F. E., et al. (2021). "Effectiveness of an Interpersonal Psychotherapy (IPT) Group Depression Treatment for Head Start Mothers: A Cluster-Randomized Controlled Trial." J Affect Disord 280(Pt B): 39-48. |
| Michalak, 2015 | Michalak J, Schultze M, Heidenreich T, Schramm E. A randomized controlled trial on the efficacy of mindfulness-based cognitive therapy and a group version of cognitive behavioral analysis system of psychotherapy for chronically depressed patients. Journal of Consulting and Clinical Psychology. 2015;83(5):951-63. |
| Milgrom, 2015a | Milgrom J, Gemmill AW, Ericksen J, Burrows G, Buist A, Reece J. Treatment of postnatal depression with cognitive behavioural therapy, sertraline and combination therapy: A randomised controlled trial. Australian and New Zealand Journal of Psychiatry. 2015;49(3):236-45. |
| Milgrom, 2015b | Milgrom J, Holt C, Holt CJ, Ross J, Ericksen J, Gemmill AW. Feasibility study and pilot randomised trial of an antenatal depression treatment with infant follow-up. Archives of Women's Mental Health. 2015;18(5):717-30. |
| Milgrom, 2016 | Milgrom J, Danaher BG, Gemmill AW, Holt C, Holt CJ, Seeley JR, et al. Internet cognitive behavioral therapy for women with postnatal depression: A randomized controlled trial of MumMoodBooster. Journal of Medical Internet Research. 2016;18(3):e54. |
| Milgrom, 2021 | Milgrom, J., et al. (2021). "Internet and Face-to-face Cognitive Behavioral Therapy for Postnatal Depression Compared With Treatment as Usual: Randomized Controlled Trial of MumMoodBooster." J Med Internet Res 23(12): e17185. |
| Miranda, 2003 | Miranda J, Chung JY, Green BL, Krupnick J, Siddique J, Revicki DA, et al. Treating depression in predominantly low-income young minority women: A randomized controlled trial. Jama. 2003;290(1):57-65. |
| Misri, 2004 | Misri S, Reebye P, Corral M, Milis L. The use of paroxetine and cognitive-behavioral therapy in postpartum depression and anxiety: A randomized controlled trial. Journal of Clinical Psychiatry. 2004;65(9):1236-41. |
| Mitchell, 2009 | Mitchell PH, Veith RC, Becker KJ, Buzaitis A, Cain KC, Fruin M, et al. Brief psychosocial–behavioral intervention with antidepressant reduces poststroke depression significantly more than usual care with antidepressant living well with stroke: Randomized, controlled trial. Stroke; a journal of cerebral circulation. 2009;40(9):3073-8. |
| Mohr, 2000 | Mohr DC, Likosky W, Bertagnolli A, Goodkin DE, Van Der Wende J, Dwyer P, et al. Telephone-administered cognitive-behavioral therapy for the treatment of depressive symptoms in multiple sclerosis. Journal of Consulting and Clinical Psychology. 2000;68(2):356-61. |
| Mohr, 2011 | Mohr DC, Carmody T, Erickson L, Jin L, Leader J. Telephone-administered cognitive behavioral therapy for veterans served by community-based outpatient clinics. Journal of Consulting and Clinical Psychology. 2011;79(2):261-5. |
| Mohr, 2013 | Mohr DC, Duffecy J, Ho J, Kwasny M, Cai X, Burns MN, et al. A randomized controlled trial evaluating a manualized TeleCoaching protocol for improving adherence to a web-based intervention for the treatment of depression. PLoS One. 2013;8(8):e70086. |
| Moldovan, 2013 | Moldovan R, Cobeanu O, David D. Cognitive bibliotherapy for mild depressive symptomatology: Randomized clinical trial of efficacy and mechanisms of change. Clinical Psychology and Psychotherapy. 2013;20(6):482-93. |
| Mossey, 1996 | Mossey JM, Knott KA, Higgins M, Talerico K. Effectiveness of a psychosocial intervention, interpersonal counseling, for subdysthymic depression in medically ill elderly. The journals of gerontology Series A, Biological sciences and medical sciences. 1996;51(4):M172-8. |
| Mukhtar, 2011 | Mukhtar, F., Oei, T. P., & Yaacob, M. (2011). Effectiveness of group cognitive behaviour therapy augmentation in reducing negative cognitions in the treatment of depression in Malaysia. ASEAN Journal of Psychiatry, 12(1), 50-65. |
| Mulcahy, 2010 | Mulcahy R, Reay RE, Wilkinson RB, Owen C. A randomised control trial for the effectiveness of group interpersonal psychotherapy for postnatal depression. Archives of Women's Mental Health. 2010;13(2):125-39. |
| Murphy, 1984 | Murphy GE, Simons AD, Wetzel RD, Lustman PJ. Cognitive therapy and pharmacotherapy. Singly and together in the treatment of depression. Archives of General Psychiatry. 1984;41(1):33-41. |
| Mynors-Wallis, 1995 | Mynors-Wallis L, Gath D, Lloyd-Thomas A, Tomlinson D. Randomised controlled trial comparing problem solving treatment with amitriptyline and placebo for major depression in primary care. BMJ. 1995;310(6977):441-5. |
| Mynors-Wallis, 2000 | Mynors-Wallis LM, Gath DH, Day A, Baker F. Randomised controlled trial of problem solving treatment, antidepressant medication, and combined treatment for major depression in primary care. BMJ. 2000;320(7226):26-30. |
| Naeem, 2011 | Naeem, 2011 |
| Nakagawa, 2017 | Nakagawa, A., Mitsuda, D., Sado, M., Abe, T., Fujisawa, D., Kikuchi, T., . . . Ono, Y. (2017). Effectiveness of supplementary cognitive-behavioral therapy for pharmacotherapy-resistant depression: A randomized controlled trial. Journal of clinical psychiatry, 78(8), 1126-1135 |
| Nakimuli-Mpungu, 2015 | Nakimuli-Mpungu E, Wamala K, Okello J, Alderman S, Odokonyero R, Mojtabai R, et al. Group support psychotherapy for depression treatment in people with HIV/AIDS in northern Uganda: A single-centre randomised controlled trial. The Lancet HIV. 2015;2(5):e190-e9. |
| Nakimuli-Mpungu, 2020 | Nakimuli-Mpungu E, Musisi S, Wamala K, et al. Effectiveness and cost-effectiveness of group support psychotherapy delivered by trained lay health workers for depression treatment among people with HIV in Uganda: a cluster-randomised trial. Lancet Glob Health. 2020;8(3):e387-e398. |
| Nasrin, 2017 | Nasrin, F., Rimes, K., Reinecke, A., Rinck, M., & Barnhofer, T. (2017). Effects of Brief Behavioural Activation on Approach and Avoidance Tendencies in Acute Depression: preliminary Findings. Behavioural and Cognitive Psychotherapy, 45(1), 58-72. |
| Newby, 2017 | Newby, J., Robins, L., Wilhelm, K., Smith, J., Fletcher, T., Gillis, I., . . . Andrews, G. (2017). Web-Based Cognitive Behavior Therapy for Depression in People With Diabetes Mellitus: a Randomized Controlled Trial. Journal of medical Internet research, 19(5), e157. |
| Nezu, 1986 | Nezu AM. Efficacy of a social problem-solving therapy approach for unipolar depression. Journal of Consulting and Clinical Psychology. 1986;54(2):196-202. |
| Nezu, 1989 | Nezu AM, Perri MG. Social problem-solving therapy for unipolar depression: An initial dismantling investigation. Journal of Consulting and Clinical Psychology. 1989;57(3):408-13. |
| Ngai, 2015 | Ngai FW, Wong P-C, Leung KY, Chau PH, Chung KF. The effect of telephone-based cognitive-behavioral therapy on postnatal depression: A randomized controlled trial. Psychotherapy and Psychosomatics. 2015;84(5):294-303. |
| Niedermoser, 2020 | Niedermoser DW, Kalak N, Kiyhankhadiv A, et al. Workplace-Related Interpersonal Group Psychotherapy to Improve Life at Work in Individuals With Major Depressive Disorders: a Randomized Interventional Pilot Study. Frontiers in psychiatry. 2020;11. |
| O'Neil, 2014 | O'Neil A, Taylor B, Sanderson K, Cyril S, Chan B, Hawkes AL, et al. Efficacy and feasibility of a tele-health intervention for acute coronary syndrome patients with depression: Results of the "MoodCare" randomized controlled trial. Annals of Behavioral Medicine. 2014;48(2):163-74. |
| Oehler, 2020 | Oehler C, Görges F, Rogalla M, Rummel-Kluge C, Hegerl U. Efficacy of a Guided Web-Based Self-Management Intervention for Depression or Dysthymia: Randomized Controlled Trial With a 12-Month Follow-Up Using an Active Control Condition. J Med Internet Res. 2020;22(7):e15361. |
| Olukolade, 2017 | Olukolade, O., & Osinowo, H. (2017). Efficacy of Cognitive Rehabilitation Therapy on Poststroke Depression among Survivors of First Stroke Attack in Ibadan, Nigeria. Behavioural Neurology, 2017 |
| Omidi, 2013 | Omidi A, Mohammadkhani P, Mohammadi A, Zargar F. Comparing mindfulness based cognitive therapy and traditional cognitive behavior therapy with treatments as usual on reduction of major depressive disorder symptoms. Iranian Red Crescent Medical Journal. 2013;15(2):142-6. |
| Pagoto, 2013 | Pagoto S, Schneider KL, Whited MC, Oleski JL, Merriam P, Appelhans B, et al. Randomized controlled trial of behavioral treatment for comorbid obesity and depression in women: The Be Active Trial. International Journal of Obesity. 2013;37(11):1427-34. |
| Pecheur, 1984 | Pecheur DR, Edwards KJ. A comparison of secular and religious versions of cognitive therapy with depressed Christian college students. Journal of Psychology and Theology. 1984. |
| Peden, 2000 | Peden AR, Hall LA, Rayens MK, Beebe LL. Reducing negative thinking and depressive symptoms in college women. Journal of Nursing Scholarship. 2000;32(2):145-51. |
| Pellas, 2022 | Pellas, J., Renner, F., Ji, J. L., & Damberg, M. (2022). Telephone-based behavioral activation with mental imagery for depression: A pilot randomized clinical trial in isolated older adults during the Covid-19 pandemic. Int J Geriatr Psychiatry, 37(1). doi:10.1002/gps.5646 |
| Perini, 2009 | Perini S, Titov N, Andrews G. Clinician-assisted Internet-based treatment is effective for depression: Randomized controlled trial. Australian and New Zealand Journal of Psychiatry. 2009;43(6):571-8. |
| Poleshuck, 2014 | Poleshuck EL, Gamble SA, Bellenger K, Lu N, Tu X, Sorensen S, et al. Randomized controlled trial of interpersonal psychotherapy versus enhanced treatment as usual for women with co-occurring depression and pelvic pain. Journal of psychosomatic research. 2014;77(4):264-72. |
| Pot, 2010 | Pot AM, Bohlmeijer ET, Onrust S, Melenhorst A-S, Veerbeek M, De Vries W. The impact of life review on depression in older adults: A randomized controlled trial. International Psychogeriatrics. 2010;22(4):572-81. |
| Pots, 2014 | Pots WTM, Meulenbeek PAM, Veehof MM, Klungers J, Bohlmeijer ET. The efficacy of mindfulness-based cognitive therapy as a public mental health intervention for adults with mild to moderate depressive symptomatology: A randomized controlled trial. PLoS One. 2014;9(10). |
| Pots, 2016 | Pots WT, Fledderus M, Meulenbeek PA, ten Klooster PM, Schreurs KM, Bohlmeijer ET. Acceptance and commitment therapy as a web-based intervention for depressive symptoms: Randomised controlled trial. The British Journal of Psychiatry. 2016;208(1):69-77. |
| Pott, 2022 | Pott, S. L., Kellett, S., Green, S., Daughters, S., & Delgadillo, J. (2022). Behavioral activation for depression delivered by drug and alcohol treatment workers: A pilot randomized controlled trial. J Subst Abuse Treat, 139, 108769. doi:10.1016/j.jsat.2022.108769 |
| Preschl, 2012 | Preschl B, Maercker A, Wagner B, Forstmeier S, Banos RM, Alcaniz M, et al. Life-review therapy with computer supplements for depression in the elderly: A randomized controlled trial. Aging and Mental Health. 2012;16(8):964-74. |
| Propst, 1992 | Propst LR, Ostrom R, Watkins P, Dean T, Mashburn D. Comparative efficacy of religious and nonreligious cognitive-behavioral therapy for the treatment of clinical depression in religious individuals. Journal of Consulting and Clinical Psychology. 1992;60(1):94-103. |
| Psarraki, 2021 | Psarraki, E. E., et al. (2021). "The effects of Pythagorean Self-Awareness Intervention on patients with major depressive disorder: A pilot randomized controlled trial." J Psychiatr Res 138: 326-334. |
| Qiu, 2013 | Qiu J, Chen W, Gao X, Xu Y, Tong H, Yang M, et al. A randomized controlled trial of group cognitive behavioral therapy for Chinese breast cancer patients with major depression. Journal of psychosomatic obstetrics and gynaecology. 2013;34(2):60-7. |
| Raevuori, 2021 | Raevuori, A., et al. (2021). "A therapist-guided smartphone app for major depression in young adults: A randomized clinical trial." J Affect Disord 286: 228-238. |
| Ravindran, 1999 | Ravindran AV, Anisman H, Merali Z, Charbonneau Y, Telner J, Bialik RJ, et al. Treatment of primary dysthymia with group cognitive therapy and pharmacotherapy: Clinical symptoms and functional impairments. American Journal of Psychiatry. 1999;156(10):1608-17. |
| Raya-Tena, 2021 | Raya-Tena, A., et al. (2021). "Effectiveness of a Psychoeducational Group Intervention Carried Out by Nurses for Patients with Depression and Physical Comorbidity in Primary Care: Randomized Clinical Trial." Int J Environ Res Public Health 18(6). |
| Rehm, 1981 | Rehm LP, Kornblith SJ, O'Hara MW, Lamparski DM, Romano JM, Volkin JI. An evaluation of major components in a self-control therapy program for depression. Behavior modification. 1981;5(4):459-89. |
| Reins, 2019 | Reins, J. A., Boß, L., Lehr, D., Berking, M., & Ebert, D. D. (2019). The more I got, the less I need? Efficacy of Internet-based guided self-help compared to online psychoeducation for major depressive disorder. Journal of, 246, 695-705. doi:10.1016/j.jad.2018.12.065 |
| Reynolds, 1999 | Reynolds CF, 3rd, Miller MD, Pasternak RE, Frank E, Perel JM, Cornes C, et al. Treatment of bereavement-related major depressive episodes in later life: A controlled study of acute and continuation treatment with nortriptyline and interpersonal psychotherapy. American Journal of Psychiatry. 1999;156(2):202-8. |
| Richards, 2015 | Richards D, Timulak L, O'Brien E, Hayes C, Vigano N, Sharry J, et al. A randomized controlled trial of an internet-delivered treatment: Its potential as a low-intensity community intervention for adults with symptoms of depression. Behaviour Research and Therapy. 2015;75:20-31. |
| Richards, 2018 | Richards SH, Dickens C, Anderson R, et al. Assessing the effectiveness of Enhanced Psychological Care for patients with depressive symptoms attending cardiac rehabilitation compared with treatment as usual (CADENCE): a pilot cluster randomised controlled trial. Trials 2018; 19(1). |
| Rief, 2018 | Rief W, Bleichhardt G, Dannehl K, Euteneuer F, Wambach K. Comparing the Efficacy of CBASP with Two Versions of CBT for Depression in a Routine Care Center: a Randomized Clinical Trial. Psychotherapy and psychosomatics 2018; |
| Rizvi, 2015 | Rizvi SJ, Zaretsky A, Schaffer A, Levitt A. Is immediate adjunctive CBT more beneficial than delayed CBT in treating depression?: A Pilot Study. Journal of Psychiatric Practice. 2015;21(2):107-13. |
| Rodriguez, 2011 | RodrÌguez Vega B, Palao A, Torres G, Benito G, PÈrez E, Dieguez M, et al. Combined therapy versus usual care for the treatment of depression in oncologic patients: A randomized controlled trial. Psycho-Oncology. 2011;20(9):943-52. |
| Rohan, 2007 | Rohan KJ, Roecklein KA, Lindsey KT, Johnson LG, Lippy RD, Lacy TJ, et al. A randomized controlled trial of cognitive-behavioral therapy, light therapy, and their combination for seasonal affective disorder. Journal of Consulting and Clinical Psychology. 2007;75(3):489-500. |
| Rohricht, 2013 | Rohricht F, Papadopoulos N, Priebe S. An exploratory randomized controlled trial of body psychotherapy for patients with chronic depression. Journal of Affective Disorders. 2013;151(1):85-91. |
| Ross, 1985 | Ross M, Scott M. An evaluation of the effectiveness of individual and group cognitive therapy in the treatment of depressed patients in an inner city health centre. The Journal of the Royal College of General Practitioners. 1985;35(274):239-42. |
| Rosso, 2017 | Rosso, I., Killgore, W., Olson, E., Webb, C., Fukunaga, R., Auerbach, R., . . . Rauch, S. (2017). Internet-based cognitive behavior therapy for major depressive disorder: a randomized controlled trial. Depress Anxiety, 34(3), 236-245. |
| Rude, 1986 | Rude SS. Relative benefits of assertion or cognitive self-control treatment for depression as a function of proficiency in each domain. Journal of Consulting and Clinical Psychology. 1986;54(3):390-4. |
| Russell, 2020 | Russell A, Gaunt DM, Cooper K, et al. The feasibility of low-intensity psychological therapy for depression co-occurring with autism in adults: The Autism Depression Trial (ADEPT) – a pilot randomised controlled trial. Autism. 2020;24(6):1360-1372. |
| Ruwaard, 2009 | Ruwaard J, Schrieken B, Schrijver M, Broeksteeg J, Dekker J, Vermeulen H, et al. Standardized web-based cognitive behavioural therapy of mild to moderate depression: A randomized controlled trial with a long-term follow-up. Cognitive Behaviour Therapy. 2009;38(4):206-21. |
| Ruzickova, 2021 | Ruzickova, T., et al. (2021). "P.230 Effects of online behavioural activation on depression during covid-19." European Neuropsychopharmacology 44: S36-S37. |
| Safren, 2021 | Safren, S. A., et al. (2021). "Treating depression and improving adherence in HIV care with task-shared cognitive behavioural therapy in Khayelitsha, South Africa: a randomized controlled trial." J Int AIDS Soc 24(10): e25823. |
| Salamanca-Sanabria, 2020 | Salamanca-Sanabria A, Richards D, Timulak L, et al. A culturally adapted cognitive behavioral internet-delivered intervention for depressive symptoms: Randomized controlled trial. JMIR Mental Health. 2020;7(1). |
| Savari, 2021 | Savari, Y., et al. (2021). "A preliminary investigation on the effectiveness of compassionate mind training for students with major depressive disorder: A randomized controlled trial." Mindfulness 12(5): 1159-1172. |
| Schlicker, 2020 | Schlicker S, Baumeister H, Buntrock C, et al. A web- And mobile-based intervention for comorbid, recurrent depression in patients with chronic back pain on sick leave (get.back): Pilot randomized controlled trial on feasibility, user satisfaction, and effectiveness. JMIR Mental Health. 2020;7(4). |
| Schulberg, 1996 | Schulberg HC, Block MR, Madonia MJ, Scott CP, Rodriguez E, Imber SD, et al. Treating major depression in primary care practice. Eight-month clinical outcomes. Archives of General Psychiatry. 1996;53(10):913-9. |
| Scogin, 2018 | Scogin F, Lichstein K, DiNapoli EA, et al. Effects of integrated telehealth-delivered cognitive-behavioral therapy for depression and insomnia in rural older adults. Journal of psychotherapy integration 2018; 28(3): 292‐309. |
| Scott, 1997 | Scott C, Tacchi MJ, Jones R, Scott J. Acute and one-year outcome of a randomised controlled trial of brief cognitive therapy for major depressive disorder in primary care. The British Journal of Psychiatry. 1997;171:131-4. |
| Serfaty, 2009 | Serfaty MA, Haworth D, Blanchard M, Buszewicz M, Murad S, King M. Clinical effectiveness of individual cognitive behavioral therapy for depressed older people in primary care: A randomized controlled trial. Archives of General Psychiatry. 2009;66(12):1332-40. |
| Serfaty, 2020 | Serfaty M, King M, Nazareth I, et al. Effectiveness of cognitive–behavioural therapy for depression in advanced cancer: CanTalk randomised controlled trial. The British Journal of Psychiatry. 2020;216(4):213-221. |
| Serrano-Selva, 2012 | Serrano Selva JP, Latorre Postigo JM, Ros Segura L, Navarro Bravo B, Aguilar Corcoles MJ, Nieto Lopez M, et al. Life review therapy using autobiographical retrieval practice for older adults with clinical depression. Psicothema. 2012;24(2):224-9. |
| Serrano, 2004 | Serrano JP, Latorre JM, Gatz M, Montanes J. Life review therapy using autobiographical retrieval practice for older adults with depressive symptomatology. Psychology and Aging. 2004;19(2):270-7. |
| Shamsaei, 2008 | Shamsaei F, Rahimi A, Zarabian MK, Sedehi M. Efficacy of pharmacotherapy and cognitive therapy, alone and in combination in major depressive disorder. Hong Kong Journal of Psychiatry. 2008;18(2):76-80. |
| Shan, 2022 | Shan, Q., Xinxin, S., Zhijuan, X., Rongjing, D., & Minjie, Z. (2022). Effects of Cognitive Behavior Therapy on Depression, Illness Perception, and Quality of Life in Atrial Fibrillation Patients. Frontiers in Psychiatry, 13. doi:10.3389/fpsyt.2022.830363 |
| Shu, 2022 | Shu, Y., Wu, G., Bi, B., Liu, J., Xiong, J., & Kuang, L. (2022). Changes of functional connectivity of the subgenual anterior cingulate cortex and precuneus after cognitive behavioral therapy combined with fluoxetine in young depressed patients with suicide attempt. Behav Brain Res, 417, 113612. doi:10.1016/j.bbr.2021.113612 |
| Sinniah, 2017 | Sinniah, A., Oei, T., Maniam, T., & Subramaniam, P. (2017). Positive effects of Individual Cognitive Behavior Therapy for patients with unipolar mood disorders with suicidal ideation in Malaysia: a randomised controlled trial. Psychiatry research, 254, 179-189. |
| Sirey, 2005 | Sirey JA, Bruce ML, Alexopoulos GS. The treatment initiation program: An intervention to improve depression outcomes in older adults. American Journal of Psychiatry. 2005;162(1):184-6. |
| Smit, 2006 | Smit A, Kluiter H, Conradi HJ, Meer K, Tiemens BG, Jenner JA, et al. Short-term effects of enhanced treatment for depression in primary care: Results from a randomized controlled trial. Psychological Medicine. 2006;36(1):15-26. |
| Smith, 2017 | Smith, J., Newby, J. M., Burston, N., Murphy, M. J., Michael, S., Mackenzie, A., . . . Andrews, G. (2017). Help from home for depression: A randomised controlled trial comparing internet-delivered cognitive behaviour therapy with bibliotherapy for depression. Internet Interventions, 9, 25-37. |
| Songprakun, 2012 | Songprakun W, McCann TV. Evaluation of a cognitive behavioural self-help manual for reducing depression: A randomized controlled trial. Journal of psychiatric and mental health nursing. 2012;19(7):647-53. |
| Souza, 2016 | Souza L, Salum G, Mosqueiro B, Caldieraro M, Guerra T, Fleck M. Interpersonal psychotherapy as add-on for treatment-resistant depression: A pragmatic randomized controlled trial. Journal of affective disorders [Internet]. 2016; 193:[373-80 pp.]. Available from: http://onlinelibrary.wiley.com/o/cochrane/clcentral/articles/662/CN-01133662/frame.html |
| Spinelli, 2003 | Spinelli MG, Endicott J. Controlled clinical trial of interpersonal psychotherapy versus parenting education program for depressed pregnant women. American Journal of Psychiatry. 2003;160(3):555-62. |
| Spinelli, 2013 | Spinelli MG, Endicott J, Leon AC, Goetz RR, Kalish RB, Brustman LE, et al. A controlled clinical treatment trial of interpersonal psychotherapy for depressed pregnant women at 3 New York City sites. Journal of Clinical Psychiatry. 2013;74(4):393-9. |
| Spruill, 2021 | Spruill, T. M., et al. (2021). "Telephone-based depression self-management in Hispanic adults with epilepsy: a pilot randomized controlled trial." Transl Behav Med 11(7): 1451-1460. |
| Sreevani, 2013 | Sreevani R, Reddemma K, Chan CL, Leung PP, Wong V, Chan CH. Effectiveness of integrated body-mind-spirit group intervention on the well-being of Indian patients with depression: A pilot study. Journal of Nursing Research. 2013;21(3):179-86. |
| Strauss, 2012 | Strauss C, Hayward M, Chadwick P. Group person-based cognitive therapy for chronic depression: A pilot randomized controlled trial. British Journal of Clinical Psychology. 2012;51(3):345-50. |
| Strong, 2008 | Strong V, Waters R, Hibberd C, Murray G, Wall L, Walker J, et al. Management of depression for people with cancer (SMaRT oncology 1): A randomised trial. Lancet. 2008;372(9632):40-8. |
| Sugg, 2018 | Sugg HVR, Richards DA, Frost J. Morita Therapy for depression (Morita Trial): a pilot randomised controlled trial. BMJ open 2018; 8(8). |
| Swartz, 2008 | Swartz HA, Frank E, Zuckoff A, Cyranowski JM, Houck PR, Cheng Y, et al. Brief interpersonal psychotherapy for depressed mothers whose children are receiving psychiatric treatment. American Journal of Psychiatry. 2008;165(9):1155-62. |
| Szumska, 2020 | Szumska I, Gola M, Rusanowska M, et al. Mindfulness-based cognitive therapy reduces clinical symptoms, but do not change frontal alpha asymmetry in people with major depression disorder. International Journal of Neuroscience. 2020. |
| Takagaki, 2016 | Takagaki K, Okamoto Y, Jinnin R, Mori A, Nishiyama Y, Yamamura T, et al. Behavioral activation for late adolescents with subthreshold depression: A randomized controlled trial. European Child and Adolescent Psychiatry. 2016;25(11):1171-82. |
| Talbot, 2011 | Talbot NL, Chaudron LH, Ward EA, Duberstein PR, Conwell Y, O'Hara MW, et al. A randomized effectiveness trial of interpersonal psychotherapy for depressed women with sexual abuse histories. Psychiatric Services. 2011;62(4):374-80. |
| Taylor, 1977 | Taylor FG, Marshall WL. Experimental analysis of a cognitive-behavioral therapy for depression. Cognitive Therapy and Research. 1977;1(1):59-72. |
| Taylor, 2009 | Taylor CB, Conrad A, Wilhelm FH, Strachowski D, Khaylis A, Neri E, et al. Does improving mood in depressed patients alter factors that may affect cardiovascular disease risk? Journal of Psychiatric Research. 2009;43(16):1246-52. |
| Teasdale, 1984 | Teasdale JD, Fennell MJ, Hibbert GA, Amies PL. Cognitive therapy for major depressive disorder in primary care. The British Journal of Psychiatry. 1984;144:400-6. |
| Teichman, 1995 | Teichman Y, Bar-el Z, Shor H, Sirota P, Elizur A. A comparison of two modalities of cognitive therapy (individual and marital) in treating depression. Psychiatry. 1995;58(2):136-48. |
| Thompson, 2001 | Thompson LW, Coon DW, Gallagher-Thompson D, Sommer BR, Koin D. Comparison of desipramine and cognitive/behavioral therapy in the treatment of elderly outpatients with mild-to-moderate depression. The American Journal of Geriatric Psychiatry. 2001;9(3):225-40. |
| Tobin, 2017 | Tobin, K., Davey-Rothwell, M. A., Nonyane, B. A. S., Knowlton, A., Wissow, L., & Latkin, C. A. (2017). RCT of an integrated CBT-HIV intervention on depressive symptoms and HIV risk. |
| Tomasino, 2017 | Tomasino, K., Lattie, E., Ho, J., Palac, H., Kaiser, S., & Mohr, D. (2017). Harnessing Peer Support in an Online Intervention for Older Adults with Depression. American Journal of Geriatric Psychiatry, 25(10), 1109-1119. |
| Tong, 2020 | Tong, P., Bu, P., Yang, Y., Dong, L., Sun, T., & Shi, Y. (2019). Group cognitive behavioural therapy can reduce stigma and improve treatment compliance in major depressive disorder patients. Early intervention in psychiatry. doi:10.1111/eip.12841 |
| Tovote, 2014 | Tovote KA, Fleer J, Snippe E, Peeters A, Emmelkamp PMG, Sanderman R, et al. Individual mindfulness-based cognitive therapy and cognitive behavior therapy for treating depressive symptoms in patients with diabetes: Results of a randomized controlled trial. Diabetes care. 2014;37(9):2427-34. |
| Town, 2017 | Town, J., Abbass, A., Stride, C., & Bernier, D. (2017). A randomised controlled trial of Intensive Short-Term Dynamic Psychotherapy for treatment resistant depression: the Halifax Depression Study. Journal of Affective Disorders, 214, 15-25. |
| Trevillion, 2020 | Trevillion K, Ryan EG, Pickles A, et al. An exploratory parallel-group randomised controlled trial of antenatal Guided Self-Help (plus usual care) versus usual care alone for pregnant women with depression: DAWN trial. Journal of Affective Disorders. 2020;261:187-197. |
| Turner, 1979 | Turner RW, Ward MF, Turner DJ. Behavioral treatment for depression: An evaluation of therapeutic components. Journal of clinical psychology. 1979;35(1):166-75. |
| Turner, 2013 | Turner A, Hambridge J, Baker A, Bowman J, McElduff P. Randomised controlled trial of group cognitive behaviour therapy versus brief intervention for depression in cardiac patients. Australian and New Zealand Journal of Psychiatry. 2013;47(3):235-43. |
| van Bastelaar, 2011 | van Bastelaar KM, Pouwer F, Cuijpers P, Riper H, Snoek FJ. Web-based depression treatment for type 1 and type 2 diabetic patients: A randomized, controlled trial. Diabetes care. 2011;34(2):320-5. |
| van Lieshout, 2021 | Van Lieshout, R. J., et al. (2021). "Effect of Online 1-Day Cognitive Behavioral Therapy-Based Workshops Plus Usual Care vs Usual Care Alone for Postpartum Depression: A Randomized Clinical Trial." JAMA Psychiatry 78(11): 1200-1207. |
| Van Lieshout, 2022 | Van Lieshout, R. J., Layton, H., Savoy, C. D., Haber, E., Feller, A., Biscaro, A., . . . Ferro, M. A. (2022). Public Health Nurse-delivered Group Cognitive Behavioural Therapy for Postpartum Depression: A Randomized Controlled Trial. Can J Psychiatry, 67(6), 432-440. doi:10.1177/07067437221074426 |
| Vazquez-Gonzalez, 2013 | Vázquez González FL, Otero Otero P, Torres Iglesias A, Hermida Garcìa E, Blanco Seoane V, Dìaz Fernández O. A brief problem-solving indicated-prevention intervention for prevention of depression in nonprofessional caregivers. Psicothema. 2013;25(1):87-92. |
| Vazquez, 2017 | Vázquez, F. L., Torres, Á., Otero, P., Blanco, V., Díaz, O., & Estévez, L. E. (2017). Analysis of the components of a cognitive-behavioral intervention administered via conference call for preventing depression among non-professional caregivers: A pilot study. Aging Ment Health, 21(9), 938-946. |
| Verduyn, 2003 | Verduyn C, Barrowclough C, Roberts J, Tarrier N, Harrington R. Maternal depression and child behaviour problems: Randomised placebo-controlled trial of a cognitive-behavioural group intervention. British Journal of Psychiatry. 2003;183(OCT.):342-8. |
| Vigod, 2021 | Vigod, S. N., et al. (2021). "Mother matters: Pilot randomized wait‐list controlled trial of an online therapist‐facilitated discussion board and support group for postpartum depression symptoms." Depression and Anxiety 38(8): 816-825. |
| Wang, 2022 | Wang, Y., Liu, X., Peng, D., Wu, Y., Su, Y., Xu, J., . . . Fang, Y. (2022). A Preliminary Study of Different Treatment Strategies for Anxious Depression. Neuropsychiatric Disease and Treatment, 18, 11-18. doi:10.2147/NDT.S320091 |
| Watkins, 2012 | Watkins ER, Taylor RS, Byng R, Baeyens C, Read R, Pearson K, et al. Guided self-help concreteness training as an intervention for major depression in primary care: A Phase II randomized controlled trial. Psychological Medicine. 2012;42(7):1359-71. |
| Watt, 2000 | Watt LM, Cappeliez P. Integrative and instrumental reminiscence therapies for depression in older adults: Intervention strategies and treatment effectiveness. Aging and Mental Health. 2000;4(2):166-77. |
| Weissman, 1979 | Weissman MM, Prusoff BA, Dimascio A, Neu C, Goklaney M, Klerman GL. The efficacy of drugs and psychotherapy in the treatment of acute depressive episodes. American Journal of Psychiatry. 1979;136(4b):555-8. |
| Wiersma, 2014 | Wiersma JE, Schaik DJF, Hoogendorn AW, Dekker JJ, Van HL, Schoevers RA, et al. The effectiveness of the cognitive behavioral analysis system of psychotherapy for chronic depression: A randomized controlled trial. Psychotherapy and Psychosomatics. 2014;83(5):263-9. |
| Wiles, 2008 | Wiles NJ, Hollinghurst S, Mason V, Musa M, Burt V, Hyde J, et al. A randomized controlled trial of cognitive behavioural therapy as an adjunct to pharmacotherapy in primary care based patients with treatment resistant depression: A pilot study. Behavioural and Cognitive Psychotherapy. 2008;36(1):21-33. |
| Williams, 2000 | Williams JW, Barrett J, Oxman T, Frank E, Katon W, Sullivan M, et al. Treatment of dysthymia and minor depression in primary care: A randomized controlled trial in older adults. JAMA. 2000;284(12):1519-26. |
| Williams, 2013a | Williams AD, Blackwell SE, Mackenzie A, Holmes EA, Andrews G. Combining imagination and reason in the treatment of depression: A randomized controlled trial of internet-based cognitive-bias modification and internet-CBT for depression. Journal of Consulting and Clinical Psychology. 2013;81(5):793-9. |
| Williams, 2013b | Williams C, Wilson P, Morrison J, McMahon A, Andrew W, Allan L, et al. Guided self-help cognitive behavioural therapy for depression in primary care: A randomised controlled trial. PLoS One. 2013;8(1):e52735. |
| Williams, 2018 | Williams C, McClay CA, Matthews L, et al. Community-based group guided self-help intervention for low mood and stress: Randomised controlled trial. British Journal of Psychiatry 2018; 212(2): 88-95. |
| Wilson, 1983 | Wilson PH, Goldin JC, Charbonneau-Powis M. Comparative efficacy of behavioral and cognitive treatments of depression. Cognitive Therapy and Research. 1983;7(2):111-24. |
| Wollersheim, 1991 | Wollersheim JP, Wilson GL. Group treatment of unipolar depression: A comparison of coping, supportive, bibliotherapy, and delayed treatment groups. Professional Psychology: Research and Practice. 1991;22(6):496. |
| Wong, 2008a | Wong DF. Cognitive and health-related outcomes of group cognitive behavioural treatment for people with depressive symptoms in Hong Kong: Randomized wait-list control study. Australian and New Zealand Journal of Psychiatry. 2008;42(8):702-11. |
| Wong, 2008b | Wong DF. Cognitive behavioral treatment groups for people with chronic depression in Hong Kong: A randomized wait-list control design. Depression and anxiety. 2008;25(2):142-8. |
| Wong, 2018 | Wong SYS, Sun YY, Chan ATY, et al. Treating Subthreshold Depression in Primary Care: a Randomized Controlled Trial of Behavioral Activation With Mindfulness. Annals of family medicine 2018; 16(2): 111‐9. |
| Wright, 2005 | Wright JH, Wright AS, Albano AM, Basco MR, Goldsmith LJ, Raffield T, et al. Computer-assisted cognitive therapy for depression: Maintaining efficacy while reducing therapist time. American Journal of Psychiatry. 2005;162(6):1158-64. |
| Wright, 2022 | Wright, J. H., Owen, J., Eells, T. D., Antle, B., Bishop, L. B., Girdler, R., . . . Ali, S. (2022). Effect of Computer-Assisted Cognitive Behavior Therapy vs Usual Care on Depression Among Adults in Primary Care: A Randomized Clinical Trial. JAMA Netw Open, 5(2), e2146716. doi:10.1001/jamanetworkopen.2021.46716 |
| Xie, 2019 | Xie, J., He, G., Ding, S., Pan, C., Zhang, X., Zhou, J., & Iennaco, J. D. (2019). A randomized study on the effect of modified behavioral activation treatment for depressive symptoms in rural left-behind elderly. Psychotherapy research : journal of the Society for Psychotherapy Research, 29(3), 372-382. doi:10.1080/10503307.2017.1364444 |
| Yang, 2018 | Yang X, Zhao J, Chen Y, Zu S, Zhao J. Comprehensive self-control training benefits depressed college students: a six-month randomized controlled intervention trial. Journal of affective disorders 2018; 226: 251‐60. |
| Yeung, 2017 | Yeung, A., Wang, F., Feng, F., Zhang, J., Cooper, A., Hong, L., . . . Fava, M. (2017). Outcomes of an online computerized cognitive behavioral treatment program for treating chinese patients with depression: A pilot study. Asian journal of psychiatry. doi:10.1016/j.ajp.2017 |
| Zemestani, 2016 | Zemestani M, Davoodi I, Honarmand MM, Zargar Y, Ottaviani C. Comparative effects of group metacognitive therapy versus behavioural activation in moderately depressed students. Journal of Mental Health. 2016;25(6):479-85. |
| Zemestani, 2020a | Zemestani M, Mozaffari S. Acceptance and commitment therapy for the treatment of depression in persons with physical disability: a randomized controlled trial. Clin Rehabil. 2020;34(7):938-947. |
| Zu, 2014 | Zu S, Xiang Y-T, Liu J, Zhang L, Wang G, Ma X, et al. A comparison of cognitive-behavioral therapy, antidepressants, their combination and standard treatment for Chinese patients with moderate–severe major depressive disorders. Journal of Affective Disorders. 2014;152-154:262-7. |

## Appendix B. Selected characteristics of included studies

| study | adm | Ther | Ctr | frm | Ns | C | M age | Prop women | Recr | Dx | Trg grp | sg | ac | ba | itt |
| --- | --- | --- | --- | --- | --- | --- | --- | --- | --- | --- | --- | --- | --- | --- | --- |
| Ahmadpanah, 2017 3rd + ssri | 100 | 3rd | pha | grp | 8 | oth | 24.8 | 1.00 | oth | mdd | ppd | + | - | sr | + |
| Ahmadpanah, 2017 other psy +ssri | 100 | oth | pha | grp | 8 | oth | 24.8 | 1.00 | oth | mdd | ppd | + | - | + | + |
| Alexopoulos, 2016 | 37 | Pst | other | ind | 11 | us | 74.9 |  | oth | mdd | med | + | - | + | + |
| Alhusen, 2021 | 0 | Cbt | cau | grp | 5 | us | 24.5 | 1.00 | oth | cut | ppd | - | - | + | - |
| Allart van Dam, 2003 | 0 | Cbt | cau | grp | 12 | eu | 45.5 | 0.62 | com | sub | adul | - | - | sr | - |
| Ammerman, 2013 | 0 | Cbt | cau | ind | 11 | us | 21.9 | 1.00 | oth | mdd | ppd | - | + | + | + |
| Andersson, 2005 | 69 | Cbt | other | gsh | 5 | eu | 36.3 | 0.74 | com | cut | adul | + | + | sr | - |
| Arean, 1993 Irt | 0 | Lrt | wl | grp | 12 | us | 66.5 | 0.75 | com | mdd | old | - | - | + | - |
| Arean, 1993 pst | 0 | Pst | wl | grp | 12 | us | 66.5 | 0.75 | com | mdd | old | - | - | + | - |
| Arjadi, 2018 | 0 | Bat | other | gsh | 5 | oth | 24.5 | 0.81 | com | mood | adul | + | + | sr | + |
| Ashouri, 2013 3rd | 100 | 3rd | pha | ind |  | oth | 32.5 | 0.61 | clin | mdd | adul | - | - | sr | - |
| Ashouri, 2013 cbt | 100 | Cbt | pha | ind |  | oth | 32.5 | 0.61 | clin | mdd | adul | - | - | sr | - |
| Au, 2022 | 0 | Cbt | other | grp | 8 | eas |  |  | oth | sub | med | - | - | sr | - |
| Ayen, 2004 cbt | 12 | Cbt | wl | grp | 12 | eu | 51.3 | 1.00 | com | mood | oth | - | - | sr | - |
| Ayen, 2004 sup | 12 | oth | wl | grp | 12 | eu | 51.3 | 1.00 | com | mood | oth | - | - | sr | - |
| Barber, 2012 | 0 | dyn | oth | ind | 20 | us | 37.5 | 0.59 | com | mdd | adul | + | - | + | + |
| Barnhofer, 2009 | 61 | 3rd | cau | grp | 6 | uk | 41.9 | 0.68 | com | chr | adul | + | + | sr | + |
| Barrett, 2001 | 0 | pst | oth | ind | 6 | us | 44.1 | 0.64 | clin | mood | adul | + | + | + | + |
| Bastos, 2015 | 100 | dyn | pha | ind | 96 | oth | 29.6 | 0.61 | clin | mood | oth | - | - | sr | + |
| Baumeister, 2021 | 92 | cbt | cau | gsh | 6 | eu | 49.9 | 0.6 | oth | mdd | med | + | + | + | + |
| Baumgartner, 2021 -alc | 0 | cbt | wl | gsh | 4 | eu | 42.8 | 0.48 | com | cut | adul | + | + | + | + |
| Baumgartner, 2021 – alc+depr | 0 | cbt | wl | gsh | 4 | eu | 42.8 | 0.48 | com | cut | adul | + | + | + | + |
| Beach, 1992 bmt | 0 | cbt | wl | oth | 18 | us | 39.1 | 1.00 | com | mood | oth | - | - | sr | - |
| Beach, 1992 cbt | 0 | cbt | wl | ind | 18 | us | 39.1 | 1.00 | com | mood | oth | - | - | sr | - |
| Bedard, 2014 | 36 | 3rd | wl | grp | 10 | can | 46.5 | 0.45 | com | cut | med | + | + | sr | - |
| Beeber, 2010 | 0 | ipt | cau | ind | 16 | us | 26 | 1.00 | oth | cut | oth | + | - | sr | - |
| Bellack, 1981 | 100 | oth | pha | ind | 12 | us | 35.7 | 1.00 | com | mdd | adul | - | - | + | - |
| Bellino, 2006 | 100 | ipt | pha | ind | 24 | eu | 26.4 | 0.63 | clin | mdd | oth | - | - | + | - |
| Bendig, 2021 | 32 | cbt | wl | gsh | 3 | eu | 56.4 | 0.35 | oth | cut | med | + | + | + | + |
| Berger, 2011 | 25 | cbt | wl | gsh | 10 | eu | 38.8 | 0.7 | com | mood | adul | + | + | sr | + |
| Berman, 2022 | 58 | 3rd | other | grp | 11 | us | 51.4 | 1.00 | com | mdd | oth | + | - | + | + |
| Beutel, 2014 | 10 | dyn | cau | ind | 18 | eu | 51.8 | 1.00 | oth | mood | med | + | + | + | + |
| Blackburn, 1981 | 100 | cbt | pha | grp | 15 | uk | 43.3 | 0.78 | clin | mdd | adul | - | - | - | - |
| Blom, 2007 | 100 | ipt | pha | ind | 12 | eu | 40 | 0.64 | clin | mdd | adul | - | - | + | + |
| Boeie, 2018 | 11 | pst | wl | gsh | 5 | eu | 44.9 | 0.55 | com | cut | med | + | + | sr | + |
| Boeschoten, 2017 | 13 | pst | wl | gsh | 5 | eu | 48.9 | 0.8 | com | cut | med | + | + | sr | + |
| Bowman, 1995 cbt | 15,6 | cbt | wl | gsh | 4 | us | 36.2 | 0.63 | com | cut | adul | - | - | - | - |
| Bowman, 1995 pst | 15,6 | pst | wl | gsh | 4 | us | 36.2 | 0.63 | com | cut | adul | - | - | - | - |
| Browne, 2002 | 100 | ipt | pha | ind | 10 | can | 42.4 | 0.68 | com | mood | adul | + | + | + | - |
| Buntrock, 2015 | 23 |  | cau | gsh | 5 | eu | 45 | 0.74 | com | sub | adul | + | + | sr | + |
| Burnand, 2002 | 100 | dyn | pha | ind | 10 | eu | 36.4 | 0.61 | clin | mdd | adul | - | - | + | - |
| Burns, 2013 | 19 | cbt | cau | ind | 12 | uk | 29.2 | 1.00 | oth | cut | ppd | + | + | sr | + |
| Carr, 2017 | 82 | cbt | cau | grp | 20 | eu | 41 | 0.66 | clin | mdd | adul | - | - | + | + |
| Carta, 2012 | 70 | cbt | cau | ind | 12 | eu | 42.5 | 0.66 | clin | mood | adul | - | - | sr | - |
| Casanas, 2012 | 56 | cbt | cau | grp | 9 | eu | 53.4 | 0.89 | clin | mdd | adul | + | + | sr | + |
| Chan, 2012 - cbt | 100 | cbt | wl | grp | 10 | eas | 46.4 | 0.82 | clin | mdd | adul | - | + | + | - |
| Chan, 2012 - dmbi | 100 | oth | wl | grp | 10 | eas | 46.4 | 0.82 | clin | mdd | adul | - | + | + | - |
| Chesney, 2003 | 0 | oth | wl | grp | 10 | us | 39 | 0 | com | sub | med | - | - | sr | - |
| Chetty, 2013 | 100 | cbt | pha | grp | 12 | oth | 45.2 | 1.00 | clin | mood | oth | + | - | sr | + |
| Chiang, 2015 | 47 | cbt | cau | grp | 12 | eas | 46.1 | 0.63 | clin | mood | adul | + | + | + | - |
| Choi, 2020 | 100 | oth | pha | ind | 6 | eas | 32.5 | 0.68 | clin | mdd | adul | + | - | - | - |
| Choi, 2020b - bat | 50 | bat | other | oth | 5 | us | 68.7 | 0.73 | oth | cut | oth | + | - | - | + |
| Choi, 2020b - pst | 50 | pst | other | oth | 5 | us | 65.5 | 0.68 | oth | cut | oth | + | - | - | + |
| Choy, 2016 | 0 | lrt | wl | grp | 6 | eas | 78.7 | 0.65 | oth | cut | old | - | - | sr | - |
| Cohen, 2010 | 0 | oth | wl | oth | 5 | us | 43.2 | 1.00 | com | mood | oth | - | - | + | - |
| Corruble, 2016 | 100 | oth | pha | tel | 8 | eu | 46.8 | 0.7 | clin | mdd | adul | + | + | + | - |
| Cramer, 2011 | 37 | cbt | cau | grp | 12 | uk | 42.5 | 1.00 | com | cut | oth | + | - | sr | + |
| De Jong, 2018 | 49 | 3rd | wl | grp | 7 | us | 50.7 | 0.75 | com | mood | med | - | - | - | + |
| De Jonghe, 2001 | 100 | dyn | pha | ind | 16 | eu | 34 | 0.62 | clin | mdd | adul | - | - | + | + |
| De Mello, 2001 | 100 | ipt | pha | ind | 16 | oth |  | 0.8 | clin | mood | adul | - | - | + | - |
| Dekker, 2012 | 36 | cbt | cau | ind | 1 | us | 66 | 0.74 | oth | cut | med | + | + | sr | + |
| Dennis, 2020 | 0 | ipt | cau | tel | 12 | can |  | 1.00 | com | mdd | ppd | + | - | sr | - |
| Denton, 2012 | 100 | oth | pha | oth | 11 | us | 32.9 | 1.00 | com | mdd | adul | + | + | + | + |
| DeRubeis, 2005 | 0 | cbt | other | ind | 14 | us | 40 | 0.59 | com | mdd | adul | - | - | + | + |
| Desautels, 2017 | 6 | cbt | wl | ind | 8 | can | 57.1 | 1.00 | oth | cut | med | + | + | + | + |
| Dimidjian, 2006 - bat | 0 | bat | other | ind | 24 | us | 39.9 | 0.66 | com | mdd | adul | + | - | + | + |
| Dimidjian, 2006 - cbt | 0 | cbt | other | ind | 24 | us | 39.9 | 0.66 | com | mdd | adul | + | - | + | + |
| Dimidjian, 2017 | 9 | bat | cau | oth | 10 | us | 28.8 | 1.00 | oth | cut | ppd | + | + | sr | + |
| Dindo, 2012 | 47 | 3rd | wl | grp | 1 | us | 32.8 | 0.93 | oth | mdd | med | - | - | + | + |
| Dindo, 2019 | 31 | 3rd | other | grp | 1 | us | 35.7 | 0.83 | com | mdd | med | - | - | + | + |
| Dobkin, 2011 | 54 | cbt | cau | ind | 10 | us | 64.6 | 0.4 | com | mood | med | + | - | + | + |
| Dobkin, 2020 | 69 | cbt | cau | tel | 10 | us | 65.6 | 0.54 | oth | mood | med | + | + | + | + |
| Dobkin, 2021 | 49 | cbt | cau | tel | 10 | us | 66.8 | 0 | com | mdd | med | + | + | + | + |
| Doering, 2013 | 36 | cbt | cau | ind | 8 | us | 63.6 | 0.31 | oth | mood | med | + | - | sr | + |
| Dong, 2019 | 0 | lrt | cau | tel | 6 | eas | 59.1 | 0.5 | oth | cut | med | + | + | + | + |
| Dozois, 2009 | 100 | cbt | pha | ind | 15 | can | 46.5 | 0.74 | clin | mdd | adul | - | + | sr | - |
| Duarte, 2009 | 11 | cbt | cau | grp | 12 | oth | 53.2 | 0.59 | oth | mdd | med | + | + | sr | + |
| Dwight, 2011 | 32 | cbt | cau | tel | 5 | us | 39.8 | 0.78 | oth | cut | oth | + | - | sr | + |
| Ebert, 2018 | 6 | oth | wl | gsh | 5 | eu | 44.2 | 0.8 | com | sub | adul | + | + | + | + |
| Ekers, 2011 | 68 | bat | wl | ind | 12 | uk | 44.7 | 0.62 | clin | mood | adul | + | + | sr | + |
| Ekkers, 2011 | 75 | cbt | cau | grp | 7 | eu | 72.7 | 0.77 | clin | mdd | old | - | + | sr | - |
| Elkin, 1989 - cbt | 0 | cbt | other | ind | 13 | us | 35 | 0.7 | clin | mdd | adul | + | + | + | + |
| Elkin, 1989 - ipt | 0 | ipt | other | ind | 13 | us | 35 | 0.7 | clin | mdd | adul | + | + | + | + |
| Euteneuer, 2017, c | 37 | cbt | wl | ind | 16 | eu | 37.3 | 0.49 | com | mdd | adul | + | - | sr | + |
| Euteneuer, 2017 e | 37 | cbt | wl | ind | 16 | eu | 37.3 | 0.49 | com | mdd | adul | + | - | sr | + |
| Euteneuer, 2022 | 0 | cbt | wl | ind | 14 | eu | 30.3 | 0.6 | com | mdd | adul | + | - | + | + |
| Fann, 2015 - cbt-ip | 39 | cbt | cau | ind | 9 | us | 45.8 | 0.37 | com | mdd | med | + | - | + | - |
| Fann, 2015 - cbt-t | 39 | cbt | cau | tel | 10 | us | 45.8 | 0.37 | com | mdd | med | + | - | + | - |
| Finkenzeller, 2009 | 100 | ipt | pha | grp | 12 | eu | 67 | 0.5 | oth | mdd | med | + | - | + | + |
| Fledderus, 2012 - m | 0 | 3rd | wl | gsh | 9 | eu | 42 | 0.7 | com | cut | adul | + | - | sr | + |
| Fledderus, 2012 - e | 0 | 3rd | wl | gsh | 9 | eu | 42 | 0.7 | com | cut | adul | + | - | sr | + |
| Floyd, 2004 - cbt-bibl | 26 | cbt | wl | gsh | 4 | us | 68 | 0.76 | com | mood | old | - | - | - | - |
| Floyd, 2004 - cbt-ind | 26 | cbt | wl | ind | 16 | us | 68 | 0.76 | com | mood | old | - | - | - | - |
| Folke, 2012 | 79 | 3rd | cau | oth | 6 | eu | 43.2 | 0.88 | oth | mdd | oth | - | - | sr | + |
| Fonagy, 2015 | 81 | dyn | cau | ind | 60 | uk | 44.3 | 0.66 | clin | chr | adul | + | + | + | + |
| Forand, 2018 | 38 | cbt | wl | gsh | 6 | us | 33 | 0.75 | com | cut | adul | + | - | - | + |
| Forsell, 2017 | 5 | cbt | cau | gsh | 5 | eu | 31 | 1.00 | com | mdd | ppd | + | + | sr | + |
| Freedland, 2009 - cbt | 50 | cbt | cau | ind | 11 | us | 60.7 | 0.5 | oth | mood | med | + | + | + | + |
| Freedland, 2009 - sup | 50 | oth | cau | ind | 8 | us | 60.7 | 0.5 | oth | mood | med | + | + | + | + |
| Freedland, 2015 | 33 | cbt | cau | ind | 11 | us | 55.8 | 0.46 | oth | mdd | med | + | + | + | + |
| Funderburk, 2021 | 34 | bat | cau | ind | 4 | us | 53 | 0.09 | clin | cut | oth | - | + | + | + |
| Gater, 2010 | 100 | oth | pha | grp | 10 | uk | 42 | 1.00 | com | mdd | oth | + | + | + | + |
| Gaudiano, 2015 | 100 | 3rd | pha | ind | 11 | us | 50 | 0.54 | com | mdd | oth | - | - | + | + |
| Gawrysiak, 2009 | 0 | bat | cau | ind | 1 | us | 18.4 | 0.8 | com | cut | stud | - | - | sr | + |
| Gellis, 2008 | 0 | pst | cau | ind | 6 | us | 77.4 | 0.87 | oth | sub | med | + | - | + | - |
| Gellis, 2010 | 12 | pst | cau | ind | 6 | us | 75.9 | 0.92 | oth | sub | med | - | + | + | - |
| Geraedts, 2014 | 8 | cbt | cau | gsh | 6 | eu | 43.4 | 0.62 | oth | cut | oth | + | + | sr | + |
| Ghorbani, 2021 | 0 | 3rd | wl | grp | 8 | oth | 45 | 1.00 | oth | cut | med | - | - | + | - |
| Gitlin, 2013 | 19 | oth | wl | ind | 8 | us | 69.6 | 0.78 | com | cut | old | + | + | sr | + |
| Goodman, 2015 | 0 | oth | cau | ind | 8 | us | 30.7 | 1.00 | oth | cut | ppd | - | + | sr | + |
| Grote, 2009 | 0 | ipt | cau | ind | 8 | us | 24.5 | 1.00 | oth | cut | ppd | - | - | sr | + |
| Guo, 2022 | 100 | 3rd | pha | grp | 8 | eas | 37.3 |  | clin | mood | adul | - | - | - | - |
| Hagen, 2017 | 8 | 3rd | wl | ind | 10 | eu | 33.7 | 0.49 | com | mdd | adul | + | - | sr | + |
| Haliford, 2016 | 8 | lrt | cau | ind | 6 | au | 20.8 | 0.5 | clin | cut | oth | - | + | sr | + |
| Hallgren, 2015 | 28 | cbt | cau | gsh | 8 | eu | 43 | 0.73 | clin | cut | adul | + | + | + | - |
| Hamamci, 2006 - cbt | 0 | cbt | cau | grp | 11 | oth | 19.5 | 0.48 | com | cut | stud | - | - | sr | - |
| Hamamci, 2006 - cbt+drama | 0 | cbt | cau | grp | 11 | oth | 19.5 | 0.48 | com | cut | stud | - | - | sr | - |
| Haringsma, 2006 | 48 | cbt | wl | grp | 9 | eu | 64.2 | 0.69 | com | cut | old | - | - | sr | + |
| Harley, 2008 | 100 | 3rd | wl | grp | 14 | us | 41.8 | 0.75 | clin | chr | adul | - | - | + | - |
| Hautzinger, 1996 | 100 | cbt | pha | ind | 24 | eu | 38.8 | 0.63 | clin | mood | adul | - | - | + | + |
| Hautzinger, 2004 | 57 | cbt | wl | grp | 12 | eu | 68.5 | 0.79 | com | mood | old | + | - | sr | + |
| Hayman, 1980 | 0 |  | wl | grp | 8 | us | 21.3 | 1.00 | com | cut | adul | + | - | sr | - |
| He, 2019 | 62 | cbt | other | ind | 12 | eas | 30.5 | 0.5 | clin | mood | adul | - | + | + | + |
| Heckman, 2011 – cop | 25 | oth | cau | grp | 7 | us | 55.3 | 0.33 | com | cut | med | + | - | sr | + |
| Heckman, 2011 – sup | 25 | oth | cau | grp | 7 | us | 55.3 | 0.33 | com | cut | med | + | - | sr | + |
| Heckman, 2017 | 62 | ipt | cau | tel | 8 | us | 51.9 | 0.37 | com | mood | med | + | - | sr | + |
| Hegel, 2010 | 0 | cbt | other | grp | 10 | eu | 46.4 | 0.65 | clin | mood | adul | + | + | + | + |
| Hellerstein, 2001 | 100 | oth | pha | grp | 16 | us | 45.1 | 0.5 | clin | mood | adul | - | - | - | + |
| Hemanny, 2019 - bat | 100 | bat | cau | ind | 12 | oth | 39.7 | 0.86 | com | mdd | adul | + | - | + | + |
| Hemanny, 2019 - cbt | 100 | cbt | cau | ind | 12 | oth | 39.7 | 0.86 | com | mdd | adul | + | - | + | + |
| Hermanns, 2015 | 0 | cbt | cau | grp | 5 | eu | 43.3 | 0.57 | oth | sub | med | - | + | sr | + |
| Herrmann, 2016 | 12 | dyn | cau | oth | 16 | eu | 59.2 | 0.21 | oth | cut | med | + | - | sr | + |
| Hoifodt, 2013 | 19 | cbt | wl | gsh | 4 | eu | 36.1 | 0.73 | clin | cut | adul | + | - | sr | + |
| Hollon, 1992 | 100 | cbt | pha | ind | 15 | us | 32.6 | 0.8 | clin | mdd | adul | - | - | sr | + |
| Hollon, 2014 | 100 | cbt | pha | ind | 67 | us | 43.2 | 0.59 | clin | chr | adul | + | + | + | + |
| Hsiao, 2011 | 100 | oth | pha | grp | 8 | eas | 37.5 | 0.76 | clin | mdd | adul | + | - | + | + |
| Hsiao, 2014 | 69 | oth | cau | grp | 8 | eas |  | 0.71 | clin | sub | adul | + | + | sr | + |
| Hummel, 2017 | 74 | cbt | wl | grp | 13 | eu | 81.9 | 0.8 | oth | cut | med | + | - | + | + |
| Jarrett, 1999 | 0 | cbt | other | ind | 20 | us | 39.6 | 0.68 | com | mdd | adul | - | + | + | + |
| Jelinek, 2016 | 67 | 3rd | other | grp | 7 | eu | 45.5 | 0.74 | clin | mood | adul | - | - | + | + |
| Johnson, 2012 | 63 | ipt | other | oth | 27 | us | 35 | 1.00 | oth | mdd | oth | - | + | + | + |
| Johnson, 2019 | 23 | ipt | cau | oth | 24 | us | 39 | 0.35 | oth | mdd | oth | + | + | + | + |
| Kamga, 2017 | 16 | cbt | wl | gsh | 8 | can | 76 | 0.63 | oth | cut | med | + | + | sr | + |
| Keeley, 2016 | 36 | oth | cau | ind | 4 | us | 47.5 | 0.71 | clin | mdd | adul | + | + | sr | + |
| Keller, 2000 | 100 | oth | pha | ind | 16 | us | 43 | 0.65 | clin | chr | adul | + | + | + | + |
| Kenter, 2016 | 25 | pst | wl | gsh | 5 | eu | 38 | 0.54 | clin | mdd | adul | + | + | sr | + |
| Kessler, 2009 | 52 | cbt | wl | gsh | 10 | uk | 34.9 | 0.68 | clin | mood | adul | + | + | sr | + |
| Khoshbooii, 2021-grp | 0 | cbt | wl | grp | 16 | oth | 49 | 1.00 | oth | cut | oth | - | - | + | - |
| Khoshbooii, 2021 -ind | 0 | cbt | wl | ind | 8 | oth | 49 | 1.00 | oth | cut | oth | - | - | + | - |
| Kim, 2018 | 0 | cbt | cau | oth | 7 | eas | 48 | 1.00 | oth | cut | med | + | - | sr | - |
| Kramer, 2021 | 27 | cbt | wl | gsh | 6 | eu | 36.3 | 0.78 | clin | cut | adul | + | - | + | + |
| Laidlaw, 2008 | 0 | cbt | cau | ind | 8 | uk | 74 | 0.73 | clin | mdd | old | + | + | + | - |
| Lam, 2013 | 100 | cbt | pha | tel | 6 | can | 43.3 | 0.55 | com | mdd | adul | + | + | + | + |
| Lamers, 2010 | 0 | cbt | cau | ind | 4 | eu | 70.7 | 0.47 | oth | mood | med | + | + | sr | + |
| Lappalainen, 2015 | 21 | 3rd | wl | gsh | 6 | eu | 51.9 | 0.72 | com | mdd | adul | - | + | sr | + |
| Larcombe, 1984 | 16 | cbt | wl | grp | 6 | au | 42.5 | 0.68 | com | mood | med | - | - | + | - |
| Lee, 2021 | 89 | bat | cau | grp | 10 | eas | 37 | 0.58 | clin | mood | adul | + | + | + | + |
| Lenze, 2020 | 29 | ipt | cau | ind | 9 | us | 26 | 1.00 | com | mood | ppd | + | + | sr | + |
| Lerner, 2015 | 56 | cbt | cau | tel | 8 | us | 54.7 | 0.72 | oth | mood | oth | + | - | sr | + |
| Lesperance, 2007 | 100 | ipt | pha | ind | 12 | can | 58.2 | 0.25 | com | mdd | med | + | + | + | + |
| Li, 2022 | 100 | 3rd | pha | oth | 8 | eas | 67.4 | 0.77 | clin | mood | old | - | - | + | - |
| Linde, 2011 | 77 | cbt | other | grp | 11 | us | 52.2 | 1.00 | oth | cut | oth | + | + | sr | + |
| Liu, 2009 | 0 | cbt | wl | gsh | 8 | eas | 26.4 | 0.73 | com | cut | adul | - | - | sr | + |
| Lloyd-Williams, 2018 | 53 | oth | cau | ind | 1 | uk | 65.1 | 0.71 | oth | cut | med | - | + | sr | + |
| Lovell, 2008 | 69 | cbt | cau | ind | 4 | uk | 37.6 | 0.74 | clin | cut | adul | - | + | sr | + |
| Lundgren, 2016 | 18 | cbt | other | gsh | 7 | eu | 62.9 | 0.42 | oth | cut | med | + | + | sr | + |
| Lustman, 1998 | 0 | cbt | cau | ind | 10 | us | 54.8 | 0.6 | com | mdd | med | + | + | sr | + |
| Lynch, 1997 | 3 | pst | cau | tel | 6 | us | 48.3 | 0.86 | clin | sub | adul | - | - | + | - |
| Lynch, 2003 | 100 | oth | pha | oth | 56 | us | 66 | 0.85 | clin | mdd | old | - | - | - | - |
| Macaskil, 1996 | 100 | cbt | pha | ind | 20 | uk | 38.2 | 0.7 | clin | mdd | adul | - | - | sr | - |
| MacPherson, 2013 | 73 | oth | cau | ind | 8 | uk | 43.5 | 0.73 | clin | cut | adul | + | + | sr | + |
| Maina, 2005 -dyn | 0 | dyn | wl | ind | 20 | eu | 36.8 | 0.63 | clin | mood | adul | - | - | + | + |
| Maina, 2005 -sup | 0 | oth | wl | ind | 19 | eu | 36.8 | 0.63 | clin | mood | adul | - | - | + | + |
| Maina, 2010 | 100 | dyn | pha | ind | 14 | eu | 31.5 | 0.56 | clin | mdd | oth | + | + | + | + |
| Maldonado, 1984 -as | 100 | oth | pha | ind | 10 | eu |  |  | clin | mood | adul | - | - | sr | - |
| Maldonado, 1984 -cbt | 100 | cbt | pha | ind | 10 | eu |  |  | clin | mood | adul | - | - | sr | - |
| Mansour, 2022 | 0 | cbt | other | ind | 4 | oth |  | 0.69 | oth | cut | med | + | + | sr | + |
| Markowitz, 2005 | 100 | ipt | pha | ind | 10 | us | 42.3 | 0.63 | com | mood | adul | + | + | + | + |
| Matsuzaka, 2017 | 0 | ipt | cau | ind | 4 | oth | 43.8 | 0.94 | clin | mood | adul | + | + | + | + |
| McClay, 2015 | 52 | cbt | wl | grp | 4 | uk | 43.7 | 0.65 | com | cut | adul | - | + | sr | - |
| McCusker, 2021 | 23 | cbt | cau | gsh | 8 | can | 58 | 0.79 | com | cut | med | + | - | + | + |
| Mclndoo, 2016 - bat | 12 | bat | wl | ind | 4 | us | 19.2 | 0.62 | com | cut | stud | + | - | - | + |
| Mclndoo, 2016 - mbt | 12 | 3rd | wl | ind | 4 | us | 19.2 | 0.62 | com | cut | stud | + | - | - | + |
| Mennen, 2021 | 9 | ipt | cau | grp | 12 | us | 33 | 1.00 | oth | cut | ppd | + | - | + | + |
| Michalak, 2015 -cbasp | 54 | oth | cau | oth | 10 | eu | 50.8 | 0.62 | com | chr | adul | + | + | + | + |
| Michalak, 2015 -mbct | 54 | 3rd | cau | grp | 8 | eu | 50.8 | 0.62 | com | chr | adul | + | + | + | + |
| Milgrom, 2015 | 100 | cbt | pha | oth | 9 | au | 30.1 | 1.00 | oth | mood | ppd | + | + | sr | + |
| Milgrom, 2015b | 9 | cbt | cau | ind | 6 | au | 31.8 | 1.00 | com | mood | ppd | + | + | sr | + |
| Milgrom, 2016 | 0 | cbt | cau | gsh | 6 | au | 31.6 | 1.00 | com | mood | ppd | + | + | sr | + |
| Milgrom, 2021 -ftf | 9 | cbt | cau | ind | 9 | au | 32.1 | 1.00 | com | mood | ppd | + | + | + | + |
| Milgrom, 2021 -icbt | 9 | cbt | cau | gsh | 6 | au | 32.1 | 1.00 | com | mood | ppd | + | + | + | + |
| Miranda, 2003 | 0 | cbt | cau | oth | 8 | us | 29.3 | 1.00 | oth | mdd | oth | + | + | + | + |
| Misri, 2004 | 100 | cbt | pha | ind | 12 | can | 30.1 | 1.00 | clin | mood | ppd | + | - | sr | + |
| Mitchell, 2009 | 100 | oth | pha | ind | 9 | us | 57 | 0.41 | oth | mdd | med | + | + | + | + |
| Mohr, 2000 | 9 | cbt | cau | tel | 8 | us | 42.4 | 0.72 | oth | cut | med | - | - | sr | + |
| Mohr, 2011 | 41 | cbt | cau | tel | 16 | us | 55.9 | 0.09 | clin | mdd | oth | - | - | + | + |
| Mohr, 2013 | 34 | cbt | wl | gsh | 18 | us | 48.3 | 0.72 | com | mdd | adul | + | + | sr | + |
| Moldovan, 2013 | 0 | cbt | other | gsh | 4 | eu | 23 | 0.88 | com | cut | stud | + | + | sr | + |
| Mossey, 1996 | 5 | ipt | cau | ind | 10 | us | 71 | 0.78 | oth | sub | old | - | - | sr | - |
| Mukhtar, 2011 | 91 | cbt | wl | grp | 8 | eas | 40.5 | 0.55 | clin | mood | adul | - | - | sr | - |
| Mulcahy, 2010 | 54 | ipt | cau | oth | 11 | au | 32.2 | 1.00 | clin | mdd | ppd | + | - | + | - |
| Murphy, 1984 | 100 | cbt | pha | ind | 16 | us | 33.8 | 0.74 | clin | mdd | adul | + | + | sr | + |
| Mynors-Wallis, 1995 | 0 | pst | other | ind | 6 | uk | 37.1 | 0.77 | clin | mdd | adul | - | + | + | - |
| Mynors-Wallis, 2000 | 100 | pst | pha | ind | 5 | uk | 35 | 0.77 | clin | mdd | adul | + | + | + | + |
| Naeem, 2011 | 100 | cbt | pha | ind | 9 | oth | 33 | 0.74 | clin | mdd | adul | + | + | sr | + |
| Nakagawa, 2017 | 100 | cbt | cau | ind | 15 | eas | 40.6 | 0.36 | clin | chr | adul | + | + | + | + |
| Nakimuli, 2015 | 0 | oth | other | grp | 8 | oth | 44.5 | 0.42 | oth | mdd | med | + | + | sr | + |
| Nakimuli, 2020 | 0 | cbt | other | grp | 7 | oth | 38.9 | 0.55 | oth | mdd | med | + | + | - | + |
| Nasrin, 2017 | 39 | bat | wl | ind | 1 | uk | 36.3 | 0.59 | clin | mdd | adul | + | + | sr | - |
| Newby, 2017 | 40 | cbt | wl | gsh | 6 | au | 46.7 | 0.71 | com | mdd | med | + | + | sr | + |
| Nezu, 1986 - pft | 0 | pst | wl | grp | 8 | us | 41.7 | 0.77 | com | mood | adul | - | - | sr | - |
| Nezu, 1986 - pst | 0 | pst | wl | grp | 8 | us | 41.7 | 0.77 | com | mood | adul | - | - | sr | - |
| Nezu, 1989 - abbr pst | 0 | pst | wl | grp | 10 | us | 41.7 | 0.77 | com | mdd | adul | - | - | + | - |
| Nezu, 1989 - pst | 0 | pst | wl | grp | 10 | us | 41.7 | 0.77 | com | mdd | adul | - | - | + | - |
| Ngai, 2015 | 0 | cbt | cau | tel | 5 | eas | 30.8 | 1.00 | oth | cut | ppd | + | + | sr | + |
| Niedermoser, 2020 | 0 | ipt | cau | grp | 8 | eu | 40.9 | 0.5 | com | mdd | oth | + | + | - | + |
| O'Neil, 2014 | 10 | cbt | cau | tel | 8 | au | 60 | 0.25 | oth | cut | med | + | + | sr | + |
| Oehler, 2020 | 64 | cbt | other | gsh | 6 | eu | 42.9 | 0.79 | com | mood | adul | + | - | sr | + |
| Olukolade, 2017 - cbt | 0 | cbt | wl | ind | 9 | oth |  | 0.57 | oth | cut | med | + | - | sr | - |
| Olukolade, 2017 -pe | 0 | oth | wl | ind | 9 | oth |  | 0.57 | oth | cut | med | + | - | sr | - |
| Omidi, 2013 - cbt | 100 | cbt | cau | grp | 8 | oth | 32.3 | 0.67 | clin | mdd | adul | - | - | sr | - |
| Omidi, 2013 – mcbt | 100 | 3rd | cau | grp | 8 | oth | 32.3 | 0.67 | clin | mdd | adul | - | - | sr | - |
| Pagoto, 2013 | 30 | bat | other | oth | 18 | us | 45.9 | 1.00 | com | mdd | oth | + | + | + | + |
| Pecheur, 1984 -r cbt | 0 | cbt | wl | ind | 8 | us | 24 | 0.9 | com | mdd | stud | - | - | + | - |
| Pecheur, 1984 -nr cbt | 0 | cbt | wl | ind | 8 | us | 24 | 0.9 | com | mdd | stud | - | - | + | - |
| Peden, 2000 | 0 | cbt | cau | grp | 6 | us | 19.3 | 1.00 | com | cut | stud | - | - | sr | - |
| Pellas, 2022 | 13 | bat | wl | tel | 4 | eu | 75.6 | 0.83 | com | cut | old | + | + | sr | - |
| Perini, 2009 | 51 | cbt | wl | gsh | 6 | au | 49.3 | 0.78 | com | mdd | adul | + | - | sr | - |
| Poleshuck, 2014 | 21 | ipt | cau | ind | 4 | us | 36.7 | 1.00 | oth | mdd | med | + | - | - | + |
| Pot, 2010 | 0 | lrt | other | grp | 12 | eu | 64.3 | 0.73 | com | sub | old | - | + | sr | + |
| Pots, 2014 | 0 | 3rd | wl | grp | 11 | eu | 47.9 | 0.78 | com | cut | adul | + | + | sr | + |
| Pots, 2016 | 0 | 3rd | wl | gsh | 9 | eu | 46.9 | 0.76 | com | cut | adul | + | + | sr | + |
| Pott, 2022 | 47 | bat | cau | ind | 8 | uk | 42.3 | 0.27 | clin | cut | adul | + | + | sr | + |
| Preschl, 2012 | 34 | lrt | wl | ind | 6 | eu | 70 | 0.67 | com | cut | old | + | - | sr | + |
| Propst, 1992 - nrct-nt | 0 | cbt | cau | ind | 19 | us | 40 | 0.83 | com | cut | oth | - | - | + | - |
| Propst, 1992 - nrct-rt | 0 | cbt | cau | ind | 19 | us | 40 | 0.83 | com | cut | oth | - | - | + | - |
| Propst, 1992 - rct-nt | 0 | cbt | cau | ind | 19 | us | 40 | 0.83 | com | cut | oth | - | - | + | - |
| Propst, 1992 - rct-rt | 0 | cbt | cau | ind | 19 | us | 40 | 0.83 | com | cut | oth | - | - | + | - |
| Psarraki, 2021 | 32 | oth | cau | oth | 8 | eu | 47.5 | 0.84 | clin | mdd | adul | + | - | + | - |
| Qiu, 2013 | 0 | cbt | wl | grp | 10 | eas | 50.6 | 1.00 | oth | mdd | med | + | + | + | + |
| Raevuori, 2021 | 57 | oth | cau | gsh | 8 | eu | 25.1 | 0.73 | clin | mdd | stud | + | + | + | + |
| Ravindran, 1999 | 100 | cbt | pha | grp | 12 | can |  | 0.58 | com | mood | adul | + | + | + | - |
| Raya-Tena, 2021 | 60 | oth | cau | grp | 12 | eu | 68.4 | 0.82 | clin | cut | med | + | - | + | + |
| Rehm, 1981 - sm | 0 | oth | wl | grp | 7 | us | 39.2 | 1.00 | com | cut | adul | - | - | + | - |
| Rehm, 1981 - sm+se | 0 | oth | wl | grp | 7 | us | 39.2 | 1.00 | com | cut | adul | - | - | + | - |
| Rehm, 1981 sm+se+sr | 0 | oth | wl | grp | 7 | us | 39.2 | 1.00 | com | cut | adul | - | - | + | - |
| Rehm, 1981 - sm+sr | 0 | oth | wl | grp | 7 | us | 39.2 | 1.00 | com | cut | adul | - | - | + | - |
| Reins, 2019 | 20 | oth | other | gsh | 5 | eu | 41.6 | 0.76 | com | mdd | adul | + | + | + | + |
| Reynolds, 1999 | 100 | ipt | pha | ind | 8 | us | 66.1 | 0.73 | com | mdd | old | - | - | + | + |
| Richards, 2015 | 21 | cbt | wl | gsh | 7 | eu | 39.9 | 0.73 | com | cut | adul | - | + | sr | + |
| Richards, 2018 | 0 | bat | cau | ind | 8 | uk | 65.3 | 0.48 | oth | cut | med | + | + | sr | + |
| Rief, 2018 - cbasp | 42 | oth | wl | ind | 16 | eu | 37.5 | 0.51 | clin | mdd | adul | + | + | sr | + |
| Rief, 2018 -cbt-m | 42 | cbt | wl | ind | 16 | eu | 37.5 | 0.51 | clin | mdd | adul | + | + | sr | + |
| Rief, 2018 -cbt-e | 42 | cbt | wl | ind | 16 | eu | 37.5 | 0.51 | clin | mdd | adul | + | + | sr | + |
| Rizvi, 2015 | 89 | cbt | wl | ind | 14 | can | 42.4 | 0.74 | clin | mdd | adul | - | - | sr | - |
| Rodriguez, 2011 | 100 | oth | pha | ind | 8 | eu | 54.6 | 0.81 | oth | mood | med | + | + | + | + |
| Rohan, 2007 | 0 | cbt | wl | grp | 10 | us | 45 | 0.9 | com | mdd | adul | + | - | + | + |
| Rohricht, 2013 | 100 | oth | wl | grp | 8 | uk | 47.7 | 0.42 | clin | chr | adul | + | + | + | + |
| Ross, 1985 | 78 | cbt | wl | oth | 12 | uk | 33 | 0.63 | clin | mdd | adul | - | - | sr | - |
| Rosso, 2017 | 0 | cbt | other | gsh | 6 | us | 29 | 0.69 | com | mdd | adul | - | - | + | + |
| Rude, 1986 | 0 | oth | wl | grp | 12 | us | 40 | 1.00 | com | cut | adul | - | - | sr | - |
| Russell, 2019 | 55 | bat | cau | gsh | 8 | uk | 37.7 | 0.27 | oth | cut | oth | + | + | + | - |
| Ruwaard, 2009 | 19 | cbt | wl | gsh | 8 | eu | 42 | 0.69 | com | cut | adul | + | - | sr | + |
| Ruzickova, 2021 | 19 | bat | cau | tel | 4 | uk | 31.6 | 0.84 | com | cut | adul | - | - | + | - |
| Safren, 2021 | 6 | cbt | cau | ind | 8 | oth |  | 0.7 | oth | mdd | med | + | - | + | + |
| Salamanca, 2020 | 3 | cbt | wl | gsh | 7 | oth | 22.2 | 0.69 | oth | cut | stud | + | + | sr | - |
| Savari, 2021 | 0 | 3rd | wl | grp | 8 | oth | 24.3 | 1.00 | clin | mdd | stud | + | - | + | + |
| Schlicker, 2020 | 59 | cbt | wl | gsh | 4,8 | eu | 51.3 | 0.65 | oth | mdd | med | + | + | sr | + |
| Schramm, 2015 | 100 | oth | pha | ind | 22 | eu | 43.6 | 0.54 | clin | chr | adul | + | - | + | + |
| Schulberg, 1996 | 0 | ipt | cau | ind | 16 | us | 38.1 | 0.83 | clin | mdd | adul | - | - | + | + |
| Scogin, 2018 | 50 | cbt | other | ind | 12 | us | 75.4 | 0.83 | com | cut | old | + | + | + | + |
| Scott, 1997 | 96 | cbt | cau | ind | 6 | uk | 41 | 0.67 | clin | mdd | adul | - | - | + | - |
| Serfaty, 2009 | 28 | cbt | cau | ind | 7 | uk | 74.1 | 0.79 | com | mood | old | + | + | sr | + |
| Serfaty, 2019 | 24 | cbt | cau | ind | 5 | uk | 59.5 | 0.66 | oth | mdd | med | + | + | sr | + |
| Serrano-Selva, 2012 | 100 | lrt | other | ind | 4 | eu | 73.9 | 0.84 | clin | mdd | old | - | - | sr | + |
| Serrano, 2004 | 0 | lrt | cau | ind | 4 | eu | 77.2 | 0.77 | oth | cut | old | - | - | sr | - |
| Shamsaei, 2008 | 100 | cbt | pha | ind | 8 | oth | 36 | 0.86 | clin | mdd | adul | + | - | sr | - |
| Shan, 2022 | 69 | cbt | cau | ind | 10 | eas | 62.8 | 0.64 | oth | modd | med | + | + | + | - |
| Shu, 2022 | 100 | cbt | pha | ind | 8 | eas | 22.7 | 0.59 | clin | mdd | oth | - | - | + | - |
| Sinniah, 2017 | 100 | cbt | cau | ind | 16 | eas | 43.1 | 0.7 | clin | mood | oth | - | - | sr | + |
| Sirey, 2005 | 100 | oth | pha | ind | 5 | us | 73.2 | 0.54 | clin | mdd | old | - | - | + | + |
| Smit, 2006 | 75 | cbt | cau | ind | 14 | eu | 42.8 | 0.64 | clin | mdd | adul | + | + | sr | + |
| Smith, 2017 - i-cbt | 46 | cbt | wl | gsh | 6 | au | 39.9 | 0.82 | com | mdd | adul | + | + | sr | + |
| Songprakun, 2012 | 100 | cbt | cau | gsh | 8 | eas | 42.1 | 0.73 | clin | mdd | adul | + | + | sr | + |
| Souza, 2016 | 100 | ipt | pha | ind | 12 | oth | 49.2 | 0.85 | clin | chr | adul | + | + | + | + |
| Spinelli, 2003 | 0 | ipt | other | ind | 16 | us | 28.8 | 1.00 | com | mdd | ppd | + | - | - | - |
| Spinelli, 2013 | 0 | ipt | other | ind | 12 | us | 29.5 | 1.00 | oth | mdd | ppd | + | - | - | - |
| Spruill, 2021 | 32 | oth | cau | oth | 8 | us | 43.3 | 0.71 | oth | cut | med | + | + | + | + |
| Sreevani, 2013 | 100 | oth | cau | grp | 4 | oth | 28 | 0.6 | clin | mood | adul | + | - | sr | - |
| Strauss, 2012 | 88 | cbt | cau | grp | 9 | uk | 43 | 0.71 | clin | chr | adul | - | - | sr | + |
| Strong, 2008 | 27 | pst | cau | ind | 10 | uk | 56.6 | 0.71 | oth | mdd | med | + | + | sr | + |
| sugg, 2018 | 60 | oth | cau | ind | 8 | uk | 49.2 | 0.61 | com | mdd | adul | + | + | sr | + |
| Swartz, 2008 | 21 | ipt | cau | ind | 9 | us | 42.7 | 1.00 | com | mdd | oth | - | - | - | - |
| Szumska, 2020 | 0 | 3rd | wl | grp | 8 | eu | 32.4 | 0.67 | com | mdd | adul | - | - | sr | - |
| Takagaki, 2016 | 0 | bat | cau | grp | 5 | eas | 18.2 | 0.38 | oth | sub | stud | + | + | sr | + |
| Talbot, 2011 | 61 | ipt | cau | ind | 13 | us | 36 | 1.00 | clin | mdd | oth | - | - | - | + |
| Taylor, 1977 - bat | 0 | bat | wl | ind | 6 | can | 22.4 | 0.71 | com | cut | stud | - | - | sr | - |
| Taylor, 1977 - cbt | 0 | cbt | wl | ind | 6 | can | 22.4 | 0.71 | com | cut | stud | - | - | sr | - |
| Taylor, 1977 - ct | 0 | cbt | wl | ind | 6 | can | 22.4 | 0.71 | com | cut | stud | - | - | sr | - |
| Taylor, 2009 | 39 | cbt | wl | ind | 15 | us | 62.2 | 0.67 | com | cut | med | - | - | + | + |
| Teasdale, 1984 | 68 | cbt | cau | ind | 15 | uk | 37.5 | 0.94 | clin | mdd | adul | - | - | sr | - |
| Teichman, 1995 - ind | 40 | cbt | wl | ind | 13 | oth | 47.9 | 0.53 | clin | mood | adul | - | - | sr | - |
| Teichman, 1995 - mar | 40 | cbt | wl | oth | 13 | oth | 47.9 | 0.53 | clin | mood | adul | - | - | sr | - |
| Thompson, 2001 | 100 | cbt | pha | ind | 18 | us | 66.8 | 0.67 | com | mdd | old | - | - | - | + |
| Tobin, 2017 | 19 | cbt | other | oth | 10 | us | 43.6 | 0.43 | com | cut | med | + | + | sr | + |
| Tomasino, 2017 - icbt | 42 | cbt | wl | gsh | 11 | us | 69.6 | 0.68 | com | cut | old | - | - | sr | - |
| Tomasino, 2017 +peer | 42 | cbt | wl | gsh | 11 | us | 69.6 | 0.68 | com | cut | old | - | - | sr | - |
| Tong, 2019 | 100 | cbt | wl | grp | 8 | eas | 37.7 | 0.72 | clin | mdd | adul | + | - | + | - |
| Tovote, 2014 - cbt | 11 | cbt | wl | ind | 8 | eu | 53.1 | 0.49 | oth | cut | med | + | - | - | + |
| Tovote, 2014 - mbct | 11 | 3rd | wl | ind | 8 | eu | 53.1 | 0.49 | oth | cut | med | + | - | - | + |
| Town, 2017 | 100 | dyn | cau | ind | 16 | can | 41.6 | 0.63 | clin | chr | adul | + | + | + | + |
| Trevillion, 2020 | 0 | cbt | cau | oth | 6,5 | uk |  | 1.00 | com | mood | ppd | + | + | sr | + |
| Turner, 1979 | 0 | bat | other | ind | 5 | us | 24.5 | 0.5 | com | cut | adul | - | - | sr | - |
| Turner, 2013 | 39 | cbt | other | grp | 6 | au | 62 | 0.26 | com | cut | med | + | + | sr | - |
| van Bastelaar, 2011 | 11 | cbt | wl | gsh | 8 | eu | 50 | 0.61 | com | cut | med | + | + | sr | + |
| Van Lieshout, 2021 | 22 | cbt | wl | oth | 1 | can | 31.8 | 1.00 | com | cut | ppd | + | + | + | + |
| van Lieshout, 2022 | 24 | cbt | cau | grp | 9 | can | 30.9 | 1.00 | com | cut | ppd | + | + | sr | + |
| Vazquez, 2013 | 0 | pst | cau | grp | 5 | eu | 53.9 | 1.00 | oth | cut | oth | - | + | sr | + |
| Vazquez, 2017 - bat | 0 | bat | cau | oth | 4 | eu | 58.4 | 0.93 | oth | sub | oth | + | + | sr | + |
| Vazquez, 2017 - cbt | 0 | cbt | cau | oth | 5 | eu | 58.4 | 0.93 | oth | sub | oth | + | + | sr | + |
| Verduyn, 2003 - cbt | 37 | cbt | cau | grp | 16 | uk | 29.8 | 1.00 | oth | cut | oth | - | + | + | - |
| Verduyn, 2003 - sup | 37 | oth | cau | grp | 16 | uk | 29.8 | 1.00 | oth | cut | oth | - | + | + | - |
| Vigod, 2021 | 31 | ipt | wl | grp | 10 | can | 33 | 1.00 | com | cut | ppd | - | - | + | + |
| Wang, 2022 | 100 | cbt | pha | ind | 10 | eas | 28 | 0.73 | clin | mdd | adul | + | + | - | - |
| Watkins, 2012 | 49 | oth | cau | oth | 7 | uk | 43.6 | 0.64 | clin | mood | adul | + | + | + | + |
| Watt, 2000 - Irt-instr | 0 | lrt | other | grp | 6 | can | 68.6 | 0.54 | com | cut | old | - | - | + | - |
| Watt, 2000 - Irt-integr | 0 | lrt | other | grp | 6 | can | 68.6 | 0.54 | com | cut | old | - | - | + | - |
| Weissman, 1979 -ipt | 100 | ipt | pha | ind | 16 | us |  |  | clin | mdd | adul | - | - | + | - |
| Weissman, 1979 -com | 100 | ipt | pla | ind | 16 | us |  |  | clin | mdd | adul | - | - | + | - |
| Wiersma, 2014 | 64 | oth | cau | ind | 24 | eu | 41.6 | 0.6 | clin | chr | adul | + | + | sr | + |
| Wiles, 2013 | 100 | cbt | pha | ind | 11 | uk | 49.6 | 0.74 | clin | chr | adul | + | + | sr | + |
| Williams, 2000 | 0 | pst | other | ind | 6 | us | 71 | 0.41 | com | mood | old | + | + | sr | + |
| Williams, 2013a | 43 | cbt | wl | gsh | 13 | au | 44.8 | 0.76 | com | mdd | adul | + | + | sr | + |
| Williams, 2013b | 58 | cbt | cau | gsh | 2 | uk | 41.8 | 0.68 | clin | cut | adul | - | + | sr | + |
| Williams, 2018 | 49 | cbt | wl | grp | 8 | uk | 46.6 | 0.68 | com | cut | adul | + | + | sr | + |
| Wilson, 1983 - bat | 8 | bat | wl | ind | 8 | au | 39.5 | 0.8 | com | cut | adul | - | - | - | - |
| Wilson, 1983 - cbt | 8 | cbt | wl | ind | 8 | au | 39.5 | 0.8 | com | cut | adul | - | - | - | - |
| Wollersheim, 1991 - cbt | 0 | cbt | wl | grp | 10 | us | 39.4 | 0.72 | com | mdd | adul | - | - | sr | - |
| Wollersheim, 1991 - cbt-bibl | 0 | cbt | wl | gsh | 10 | us | 39.4 | 0.72 | com | mdd | adul | - | - | sr | - |
| Wollersheim, 1991 - sup | 0 | oth | wl | grp | 10 | us | 39.4 | 0.72 | com | mdd | adul | - | - | sr | - |
| Wong, 2008a | 65 | cbt | wl | grp | 10 | eas | 42.7 | 0.78 | com | cut | adul | - | + | sr | + |
| Wong, 2008b | 100 | cbt | wl | grp | 10 | eas | 37.4 | 0.78 | com | mdd | adul | - | + | sr | - |
| Wong, 2018 | 0 | 3rd | cau | grp | 8 | eas | 54 | 0.93 | clin | sub | adul | + | + | sr | + |
| Wright, 2005 - c-cbt | 0 | cbt | wl | gsh | 9 | us | 40.2 | 0.76 | com | mdd | adul | - | - | + | - |
| Wright, 2005 - cbt-ftf | 0 | cbt | wl | ind | 9 | us | 40.2 | 0.76 | com | mdd | adul | - | - | + | - |
| Wright, 2022 | 24 | cbt | cau | gsh | 9 | us | 47 | 0.85 | clin | cut | adul | + | + | sr | + |
| Xie, 2019 | 0 | bat | cau | grp | 8 | eas | 71.9 | 0.59 | oth | cut | old | + | - | sr | - |
| Yang, 2018 | 0 | oth | cau | grp | 11 | eas | 18.5 | 0.6 | oth | mdd | stud | + | - | sr | - |
| Yeung, 2017 | 32 | cbt | wl | gsh | 5 | eas | 33 | 0.77 | clin | cut | adul | + | + | sr | - |
| Zemestani, 2016 - bat | 0 | bat | wl | grp | 8 | oth | 24.2 | 0.61 | oth | mdd | stud | + | - | 2 | + |
| Zemestani, 2016 - mct | 0 | 3rd | wl | grp | 8 | oth | 24.2 | 0.61 | oth | mdd | stud | + | - | 2 | + |
| Zemestani, 2019 | 0 | 3rd | cau | grp | 8 | oth | 29.6 | 1.00 | oth | mood | ppd | + | + | 2 | + |
| zu, 2014 – cbt | 0 | cbt | cau | ind | 20 | eas | 38.5 | 0.51 | clin | mdd | adul | - | - | - | - |
| Zu, 2014 - cbt + ssri | 100 | cbt | pha | ind | 20 | eas | 38.5 | 0.51 | clin | mdd | adul | + | - | + | - |
|  |  |  |  |  |  |  |  |  |  |  |  |  |  |  |  |

Abbreviations: 3rd: Third wave therapy; Ac: allocation concealment; Adm: proportion users of antidepressants; adul: adults; Au: Australia; Ba: blinded assessment; Bat: behavioral activation therapy; C: Country; Can: Canada; Cau: care-as-usual; Cbt: cognitive behavior therapy; chr: chronic depression; clin: clinical; com: community; Ctr: type of control group; cut: scoring above a cut-off; Dx: Diagnosis; Dyn: psychodynamic therapy; Eas: East Asia; Eu: Europe; Frm: Format; Grp: group; gsh: guided self-help; Ind: individual; Ipt: interpersonal psychotherapy; Itt: intention-to-treat analyses; Lrt: life review therapy; M age: Mean age; mdd: major depressive disorder; med: general medical patients; mood: mood disorder; Ns: Number of sessions; old: older adults; Oth: other; Pha: pharmacotherapy; ppd: perinatal depression; Prop women: proportion women; Pst: problem-solving therapy; Recr: Recruitment; Sg: sequence generation; sr: self-report; stud: students; sub: subthreshold depression; Sup: non-directive supportive counseling; Tel: telephone; Ther: Type of therapy; Trg grp: Target group; Uk: United Kingdom; Us: United States; Wl: waitlist.

## Appendix C. Regression models of antidepressant use in trials comparing psychotherapy with control groups with baseline severity included

|  |  | Coef. | SE | p |
| --- | --- | --- | --- | --- |
| Proportion antidepressant users (continuous) | | -0.00 | 0.00 | 0.95 |
| Baseline severity | | 0.06 | 0.02 | <0.001 |
| Therapy | Third wave therapies | Ref |  |  |
|  | Behavioral activation | 0.26 | 0.33 | 0.44 |
|  | Cognitive behavior therapy | 0.28 | 0.24 | 0.24 |
|  | Interpersonal psychotherapy | -0.22 | 0.28 | 0.42 |
|  | Problem-solving therapy | 0.44 | 0.41 | 0.29 |
|  | Life review therapy | 0.87 | 0.72 | 0.23 |
|  | Psychodynamic therapy | 0.22 | 0.41 | 0.60 |
|  | Other | 0.08 | 0.26 | 0.76 |
| Control | Care-as-usual | Ref |  |  |
|  | Pharmacotherapy | -0.07 | 0.24 | 0.78 |
|  | Waiting list | 0.52 | 0.17 | <0.01 |
|  | Other control | 0.35 | 0.22 | 0.12 |
| Year (continuous) | | 0.00 | 0.01 | 0.57 |
| Format | Group | Ref |  |  |
|  | Guided self-help | -0.49 | 0.22 | 0.03 |
|  | Individual | -0.20 | 0.15 | 0.20 |
|  | Telephone | 0.08 | 0.33 | 0.82 |
|  | Other/mixed | -0.19 | 0.23 | 0.41 |
| Number of sessions (continuous) | | -0.01 | 0.01 | 0.31 |
| Country | Australia | Ref |  |  |
|  | Canada | -0.30 | 0.32 | 0.35 |
|  | East Asia | 0.26 | 0.37 | 0.48 |
|  | Europe | -0.10 | 0.29 | 0.74 |
|  | United Kingdom | -0.21 | 0.31 | 0.50 |
|  | United States | -0.14 | 0.27 | 0.61 |
|  | Other | 0.24 | 0.31 | 0.45 |
| Mean age (continuous) | | 0.01 | 0.01 | 0.38 |
| Proportion women (continuous) | | 0.31 | 0.35 | 0.38 |
| Recruitment | Clinical |  |  |  |
|  | Community | 0.02 | 0.15 | 0.89 |
|  | Other | 0.09 | 0.21 | 0.66 |
| Diagnosis | Depressive disorder | 0.11 | 0.15 | 0.46 |
|  | Cut-off | Ref |  |  |
|  | Subthreshold depression | 0.27 | 0.39 | 0.49 |
| Target group | Adults | Ref |  |  |
|  | General medical patients | 0.03 | 0.27 | 0.91 |
|  | Older adults | -0.23 | 0.48 | 0.63 |
|  | Perinatal depression | 0.31 | 0.28 | 0.26 |
|  | Students | 0.47 | 0.32 | 0.15 |
|  | Other | 0.14 | 0.18 | 0.44 |
| Low Risk of Bias | Low vs other | -0.33 | 0.14 | 0.02 |

Abbreviations: Coef: coefficient; p: p-value; Ref: reference category; SE: standard error.

## Appendix D. Regression models of antidepressant use in trials comparing psychotherapy with control groups: Zero versus all participants using antidepressants

|  |  | Coef | SE | p |  | Coef | SE | p |  | Coef | SE | p |
| --- | --- | --- | --- | --- | --- | --- | --- | --- | --- | --- | --- | --- |
| ADM use (continuous) | | 0.00 | 0.00 | 0.03 |  | 0.00 | 0.00 | 1.00 |  | 0.00 | 0.00 | 0.66 |
| Baseline severity (continuous) | |  |  |  |  | 0.03 | 0.03 | 0.21 |  |  |  |  |
| Therapy | Third wave |  |  |  |  |  |  |  |  |  |  |  |
|  | BAT |  |  |  |  | 0.14 | 0.50 | 0.78 |  | -0.07 | 0.29 | 0.79 |
|  | CBT |  |  |  |  | 0.25 | 0.39 | 0.53 |  | -0.16 | 0.20 | 0.42 |
|  | IPT |  |  |  |  | 0.03 | 0.44 | 0.94 |  | -0.60 | 0.25 | 0.02 |
|  | PST |  |  |  |  | 0.57 | 0.52 | 0.28 |  | 0.36 | 0.29 | 0.21 |
|  | LRT |  |  |  |  | 2.22 | 1.29 | 0.09 |  | 0.31 | 0.36 | 0.40 |
|  | DYN |  |  |  |  | 0.54 | 0.53 | 0.32 |  | 0.09 | 0.33 | 0.79 |
|  | Other |  |  |  |  | 0.09 | 0.42 | 0.82 |  | -0.40 | 0.22 | 0.07 |
| Control | CAU |  |  |  |  |  |  |  |  |  |  |  |
|  | Other ctr |  |  |  |  | 0.62 | 0.41 | 0.14 |  | -0.04 | 0.19 | 0.84 |
|  | PHA |  |  |  |  | -0.04 | 0.35 | 0.90 |  | -0.28 | 0.21 | 0.19 |
|  | WL |  |  |  |  | 0.61 | 0.32 | 0.06 |  | 0.11 | 0.16 | 0.49 |
| Year (continuous) | |  |  |  |  | 0.01 | 0.01 | 0.27 |  | 0.00 | 0.01 | 0.94 |
| Format | GRP |  |  |  |  |  |  |  |  |  |  |  |
|  | GSH |  |  |  |  | -0.35 | 0.42 | 0.41 |  | 0.04 | 0.23 | 0.86 |
|  | Ind |  |  |  |  | -0.11 | 0.22 | 0.61 |  | -0.10 | 0.14 | 0.47 |
|  | Tel |  |  |  |  | 0.53 | 0.75 | 0.48 |  | 0.10 | 0.32 | 0.77 |
|  | Other/mixed |  |  |  |  | -0.84 | 0.53 | 0.12 |  | -0.16 | 0.27 | 0.54 |
| Number of sessions (continuous) | |  |  |  |  | 0.00 | 0.02 | 0.90 |  | 0.01 | 0.01 | 0.07 |
| Country | Australia |  |  |  |  |  |  |  |  |  |  |  |
|  | Can |  |  |  |  | -0.43 | 0.67 | 0.53 |  | 0.39 | 0.55 | 0.48 |
|  | East Asia |  |  |  |  | -0.58 | 0.74 | 0.44 |  | 0.10 | 0.55 | 0.86 |
|  | Europe |  |  |  |  | -0.57 | 0.67 | 0.40 |  | -0.05 | 0.54 | 0.92 |
|  | Other |  |  |  |  | 0.20 | 0.66 | 0.76 |  | 0.83 | 0.55 | 0.13 |
|  | UK |  |  |  |  | -0.06 | 0.67 | 0.93 |  | 0.00 | 0.56 | 1.00 |
|  | US |  |  |  |  | -0.40 | 0.64 | 0.53 |  | 0.15 | 0.53 | 0.78 |
| Mean age (continuous) | |  |  |  |  | 0.00 | 0.02 | 0.92 |  | 0.01 | 0.01 | 0.23 |
| Proportion women (continuous) | |  |  |  |  | 1.34 | 0.77 | 0.09 |  | 0.49 | 0.35 | 0.17 |
| Recruitment | Clinical |  |  |  |  |  |  |  |  |  |  |  |
|  | Community |  |  |  |  | -0.25 | 0.22 | 0.26 |  | -0.17 | 0.16 | 0.29 |
|  | Other |  |  |  |  | 0.08 | 0.43 | 0.86 |  | 0.09 | 0.23 | 0.71 |
| Diagnosis | Depressive dis |  |  |  |  | 0.31 | 0.26 | 0.23 |  | -0.08 | 0.16 | 0.61 |
|  | Cut-off |  |  |  |  |  |  |  |  |  |  |  |
|  | Subthreshold |  |  |  |  | 1.03 | 0.53 | 0.06 |  | -0.01 | 0.25 | 0.97 |
| Target group | Adults |  |  |  |  |  |  |  |  |  |  |  |
|  | Gen med |  |  |  |  | 0.79 | 0.65 | 0.23 |  | 0.29 | 0.25 | 0.25 |
|  | Older |  |  |  |  | 0.43 | 0.70 | 0.55 |  | -0.30 | 0.34 | 0.38 |
|  | Other |  |  |  |  | 0.35 | 0.24 | 0.15 |  | 0.44 | 0.18 | 0.01 |
|  | PPD |  |  |  |  | 0.12 | 0.56 | 0.84 |  | 0.07 | 0.30 | 0.81 |
|  | Students |  |  |  |  | 1.10 | 0.47 | 0.02 |  | 0.59 | 0.27 | 0.03 |
| Low Risk of Bias | Low vs other |  |  |  |  | -0.72 | 0.24 | 0.00 |  | -0.36 | 0.12 | 0.00 |

Abbreviations: ADM: antidepressant medication; Behav act: behavioral activation; CBT: cognitive behavior therapy; Coef: coefficient; Dis: disorder; Dynamic: psychodynamic therapy; Gen med: general medical patients; GSH: Guided self-help; IPT: interpersonal psychotherapy; k: number of comparisons; P: p-value; Perinatal: perinatal depression; Pharmacoth: pharmacotherapy; Probl. Solv:problem solving therapy; RoB: risk of bias; SE: standard error; UK: United Kingdom; US: United States.
